# *Ab initio* insights into the face, edge, and vertex interactions of BH_4_^1−^ with electron-accepting molecules

**DOI:** 10.1039/d5ra05000f

**Published:** 2025-10-15

**Authors:** Abedien Zabardasti, Mohammad Solimannejad, Mohammad N. AL-Baiati, Maryam Salehnassaj

**Affiliations:** a Department of Chemistry, Lorestan University Khorramabad Iran zabardasti.a@lu.ac.ir zebardasti.a@gmail.com; b Department of Chemistry, Faculty of Science, Arak University Arak 3848177584 Iran m-solimannejad@araku.ac.ir; c Department of Chemistry, College of Education for Pure Sciences, University of Kerbala Karbala Iraq; d Ministry of Education and Culture, Behesht Aein High School Khorramabad Iran

## Abstract

The *ab initio* calculations at the MP2/aug-cc-pvdz computational level were used to analyze the interactions of FCN, ClCN, BrCN, CF_3_H, CF_3_Cl, CH_3_OH, HF, HCl, HCN, SH_2_, SHF, SF_2_, H_2_O, HOCl, HOBr, CO, N_2,_ and H_2_ molecules with BH_4_^1−^. On BH_4_^1−^, three sites were accessible for interactions with L molecules to form BH_4_(L)^1−^ aggregates. The faces, edges, and vertices of BH_4_^1−^ as electron donors, could interact with electron acceptor species. In addition, the BH_4_^1−^ anion, through its σ-holes, could obtain electrons from interacting molecules. The significant preference of some molecules was interaction along the triangular faces, BH_4_(L)_f_^1−^ (where L = ClCN, BrCN, FCN, CF_3_Cl, CF_3_H) whereas, for others, the vertices, BH_4_(L)_v_^1−^ (where L = HOCl, HOBr, PF_3_) or edges, BH_4_(L)_e_^1−^ (where L = H_2_O, HF, HCl) of BH_4_^1−^ might be more suitable for interaction. Some molecules, such as CH_4_ and H_2_, despite their preferred facial interactions, could interplay with the vertex counterpart through an edge intermediate. It seems that accepting electrons (triel bonding) by BH_4_^1−^ σ-holes had important roles in the face interactions for BH_4_(L)_f_^1−^ adducts. Bader's Quantum Theory of Atoms in Molecules (QTAIM) and Natural Bond Orbital (NBO) calculations were used to analyze optimized complexes. Noncovalent interaction (NCI) analysis was used for further determination of interactions in BH_4_(L)^1−^ adducts.

## Abstract 2

Simple adducts, including common interactions, bi and trifurcated dihydrogen bonds in which BH_4_^1^- through its vertices, edges, and faces acts as an electron donor and through its σ-holes, acts as an electron acceptor for triel bond were considered.

## Graphical abstract

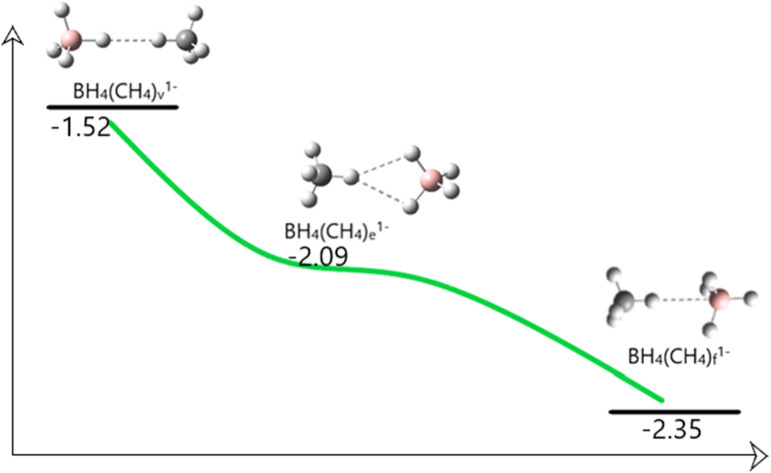

## Introduction

Boron hydrides and borane clusters are important classes of metal hydrides that have been the subject of many studies.^1–5^

Boron hydrides and borane clusters exhibit unusual bonding behavior and diverse structures, which have led to their use as ligands in inorganic chemistry and building blocks in materials science. In addition, these compounds have diverse applications stemming from their unique structures and bonding properties. These include applications in energy, materials science, and medicine. They can be used as fuels, in neutron-capture therapy for cancer treatment, and as components in polymers for heat resistance and other functional properties.^6^

Much attention has been paid to boron hydrides, thanks to their hydrogen-storage capacity, with a special emphasis on boron tetrahydride (BH_4_^1−^).^7–15^ The latter is a building block of various hydrogen-storing compounds, such as Al_3_Li_4_(BH_4_)_13_,^7^ Mg(BH_4_)_2_,^8^ Ti(BH_4_)_3_,^9^ KSc(BH_4_)_4_,^10^ Al(BH_4_)_3_,^11^ Zr(BH_4_)_4_,^12^ Hf(BH_4_)_4_,^12^ Th(BH_4_)_4_,^13^ and U(BH_4_)_4_,^13^ which can be liquid or solid-phase materials. Therefore, more detailed studies on the properties of BH_4_^1−^, especially with regard to Lewis acids and Lewis bases, are in demand. The nature and accessibility of BH_4_^1−^ as a bidentate ligand^8–13^ in various BH_4_^−1^-containing clusters can be explained by investigating its intermolecular interactions with different electron donors and electron acceptors by theoretical methods. We focused on BH_4_^1−^ as a compound that could form hydrogen-rich clusters and might be used as a hydrogen-storage system.

Previously, we studied the interaction of B_6_H_6_^2−^ with HF^14^ and H_2_.^15^ On the negatively charged surface of B_6_H_6_^2−^, the centers of the B_3_ triangular faces on the B_6_H_6_^2−^ octahedral structure, built by B–B bonds, exhibit minimal electrostatic potential. These triangular faces were electron-rich basic centers for the adsorption of H_2_ and HF molecules. In addition, other negative regions of electrostatic potentials were located on the H vertices of B_6_H_6_^2−^, but their charge densities were lower than those of the B_3_ triangles. Thus, the most significant action of H_2_ and HF molecules was interaction with the center of B_3_ triangles, which had greater charge densities.^14,15^ Similar studies using borane and carborane clusters have shown that B–B and B–C bonds could contribute as electron donors in intermolecular interactions.^16–20^

In line with those projects, the tetrahedral BH_4_^1−^ as an electron source or Lewis base, could contribute to the interactions with different kinds of electron acceptors or Lewis acids. In accordance with this idea, various types of intermolecular interactions might be considered. For this purpose, BH_4_^1−^ could be implemented in different types of interactions, including dihydrogen bonding (DHB),^21^ halogen bonding (XB),^22,23^ chalcogen bonding (ChB),^24,25^ pnictogen bonding (PnB),^26,27^ tetrel bonding (TtB),^28,29^ and triel bonding (TrB).^30^

The tetrahedral structure of BH_4_^1−^, through its B–H vertices, H–H edges, and H_3_ triangular faces, could act as an electron donor to do three types of interactions with electron-acceptor molecules. Due to the different characteristics of the electron acceptors, one would expect them to have different preferences for interaction with each one of these sites on BH_4_^1−^ (as an electron donor). Our results could aid selectivity of the interaction of several electron acceptors with an electron donor.

## Computational methods

Calculations were done using the Gaussian 09 system of codes.^31^ The geometries of the isolated BH_4_^1−^, L (where L = ClCN, BrCN, CF_3_Cl, FCN, CO, N_2,_ H_2_O, CF_3_H, CH_3_OH, HCl, HCN, HF, SH_2_, SHF, SF_2_, H_2_O, CH_3_OH, HF, HCl, HCN, CF_3_H, H_2_, HOCl, HOBr) and BH_4_(L)^1−^ complexes were fully optimized at the MP2 computational level^32^ with the aug-cc-pVDZ basis set.^33^ Harmonic vibrational frequency calculation confirmed the structures as minima, and enabled the evaluation of the zero-point energy (ZPE). The *XYZ* coordinates (*Z*-matrices) for gas-phase-optimized structures are given in Table S1 in the SI. A counterpoise procedure was used to correct the interaction energy for the basis set superposition error.^34^ AIMAll^35,36^ packages were used to obtain bond properties and molecular graphs. The NBO analysis^37^ was done employing the same method and basis set using the NBO program provided with Gaussian 09.

## Result and discussion

**Fig. 1:**
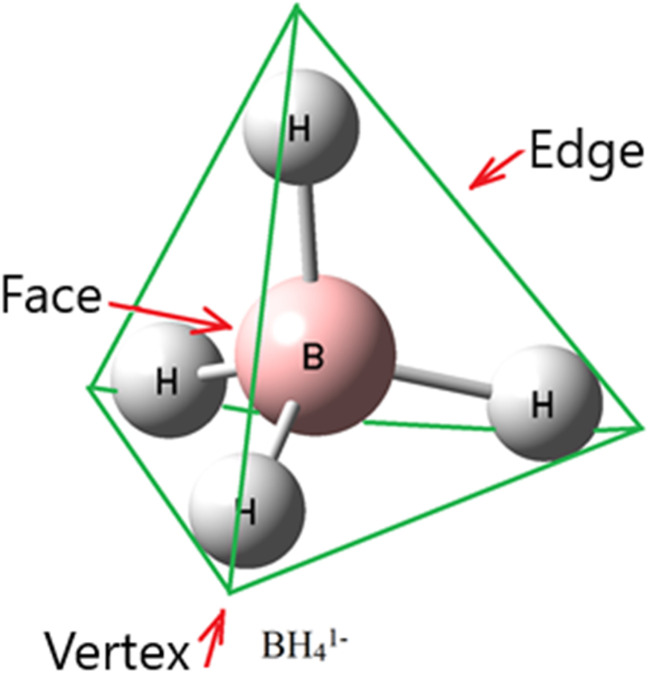
Face, vertex, and edge positions of BH_4_^1−^ complexes (schematic).

Three zones were available on BH_4_^1−^ for interactions with other molecules: H_3_ triangular faces, H_2_ edges, and BH vertices of BH_4_^1−^ (Fig. 1). The H_3_ triangular faces were present as electron acceptors for TrB and electron-donor sites for various trifurcated interactions. TrB returns to a type of interaction containing a group 13 (B, Al, Ga, *etc.*) element as an electron acceptor, forming a bond with an electron-rich region such as a lone pair, π-electron, or σ-electron.^30^ Conversely, the H_2_ edges have good conditions for contributing as electron-donor sites for some interactions like bifurcated DHB. Finally, the BH vertices of BH_4_^1−^ may contribute to conventional DHB, XB, ChB, PnB and TtB. For this purpose, BH_4_^1−^ was employed in a set of various types of interactions discussed below.

### Triel bond complexes BH_4_(L)_f_^1−^

**Fig. 2:**
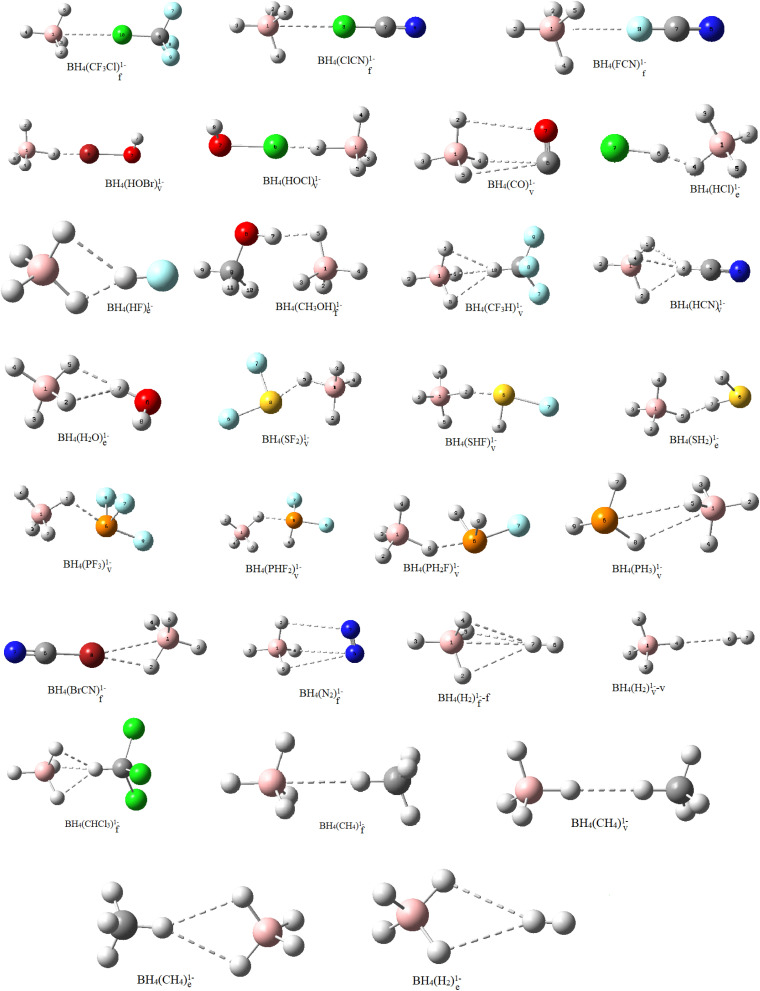
BH_4_(L)^1−^ complexes at the MP2/aug-cc-pVDZ level (schematic).

In BH_4_(FCN)_f_^1−^, BH_4_(ClCN)_f_^1−^, BH_4_(BrCN)_f_^1−^, BH_4_(ClCF_3_)_f_^1−^, BH_4_(N_2_)_f_^1−^ and BH_4_(CO)_f_^1−^ adducts, many intermolecular interactions were due to XB, PnB or TtB along with considerable L → BH_4_ charge transfers (TrB) (Fig. 2). A significant part of the interactions in these adducts returned to charge transfers from guest molecules to the σ-hole (σ*) of the B–H bonds of BH_4_^1−^, so it was termed TrB. The stabilization energies of these adducts showed the following stability (Tables 1 and S2):BH_4_(BrCN)_f_^1−^ > BH_4_(ClCN)_f_^1−^ > BH_4_(ClCF_3_)_f_^1−^ > BH_4_(FCN)_f_^1−^ > BH_4_(CO)_f_^1−^ > BH_4_(N_2_)_f_^1−^

**Table 1:** The SE^ZPE+BSSE^, Δ*H*, and Δ*G* in kcal mol^−1^ calculated at MP2/aug-cc-pVDZ^a^

Adduct	SE^ZPE+BSSE^	Δ*H*	Δ*G*	Adduct	SE^ZPE+BSSE^	Δ*H*	Δ*G*
BH_4_(ClCN)_v_^1−^	−12.74	−13.52	−8.78	BH_4_(HOBr)_v_^1−^	−20.82	−23.64	−18.65
BH_4_(BrCN)_v_^1−^	−16.28	−18.49	−12.10	BH_4_(HOCl)_v_^1−^	−16.61	−19.29	−11.15
BH_4_(CF_3_Cl)_v_^1−^	−7.13	−8.03	−2.02	BH_4_(SF_2_)_v_^1−^	−21.58	−24.58	−16.98
BH_4_(FCN)_v_^1−^	−5.96	−6.34	−2.74	BH_4_(SFH)_v_^1−^	−23.87	−26.82	−19.52
BH_4_(CO)_v_^1−^	−1.87	−2.31	2.10	BH_4_(SH_2_)_v_^1−^	−8.43	−9.79	−3.71
BH_4_(N_2_)_v_^1−^	−1.39	−1.90	1.35	BH_4_(PH_3_)_v_^1−^	−3.88	−4.59	0.86
BH_4_(H_2_O)_v_^1−^	−10.23	−11.65	−6.02	BH_4_(PH_2_F)_v_^1−^	−16.36	−18.67	−10.79
BH_4_(CH_3_OH)_v_^1−^	−11.68	−12.93	−6.89	BH_4_(PHF_2_)_v_^1−^	−15.4	−17.85	−9.65
BH_4_(HCl)_e_^1−^	−15.88	−18.19	−11.64	BH_4_(PF_3_)_v_^1−^	−12.41	−14.92	−7.46
BH_4_(HCN)_f_^1−^	−17.1	−18.38	−12.77	BH_4_(H_2_)_f_^1−^	−0.27	1.12	−4.86
BH_4_(HF)_e_^1−^	−16.07	−17.94	−12.07	BH_4_(H_2_)_v_^1−^	0.32	1.79	−5.44
BH_4_(CH_4_)_f_^1−^	−1.7	−1.94	1.94	BH_4_(H_2_)_e_^1−^*	−0.09	0.78	−4.01
BH_4_(CH_4_)_e_^1−^*	−1.48	−2.16	2.11	BH_4_(CCl_3_H)_f_^1−^	−14.15	−16.88	−9.81
BH_4_(CH_4_)_v_^1−^	−0.94	−0.90	−1.08	BH_4_(CF_3_H)_f_^1−^	−13	−14.35	−7.67

a BH_4_(CH_4_)_e_^1−^* (*v*_1_ = −48(1)) and BH_4_(H_2_)_e_^1−^* (*v*_1_ = −29(1)) are optimized nonlocal structures. SE^ZPE+BSSE^ denotes zero point- and counterpoise-corrected stabilization energies.

For BH_4_(FCN)_f_^1−^, in which the F atom seldom contributes as a halogen-bond donor, which results in halogen-bond interactions, NBO analysis indicated a partial charge of +0.0015 for the FCN molecule resulting from an NCF → BH_4_ charge transfer. More detailed analysis showed interactions of lone pairs on the F atom with the σ*_(B–H3)_ orbital (or a σ hole) of BH_4_^1−^ as TrB interactions. Therefore, the tendency to have TrB led to a face–center interaction between FCN and BH_4_^1−^. The vibrational stretching frequencies (*ν*) and lengths of B–H bonds (*r*) are given in Tables 2–4. In free BH_4_^1−^, the amount of *ν*_(B–H)_ and *r*_(B–H)_ was 2291 cm^−1^ and 1.2490 Å, respectively. These interactions caused contraction (0.0139 Å) along with a blue shift (28 cm^−1^) of the C–F bond and contraction (0.0039 Å) along with a blue shift of B–H3 (28 cm^−1^, the B–H *trans* to the FCN molecule) due to the greater contribution of B–H in adduct formation. BH_4_(FCN)_f_^1−^ showed a blue shift (3–28 cm^−1^), besides 0.0003 and 0.0039 Å reductions for its B–H bonds.

**Table 2:** Unscaled vibrational frequencies (*ν*, cm^−1^) with corresponding intensities (values given in parentheses, km mol^−1^) and bond distances (*r*, Å) for the selected bonds of free molecules

Compound	*R*	*ν*	Compound	*r*	*ν*
H_2_	0.7548	4465	SH_2_(S–H)	1.3496	2754(1)
PF_3_(P–F)	1.6294	825(203)	SHF(S–F)	1.6776	782(68)
PHF_2_(P–F)	1.6494	806(178)	SF_2_(S–F)	1.6498	798(141)
PH_2_F(P–F)	1.6727	780(120)	H_2_O(O–H)	0.9659	3804(4)
PH_3_(P–H)	1.4266	2452(34)	CH_3_OH(O–H)	0.9657	3843(44)
HOCl(Cl–O)	1.7326	740(10)	HF(H–F)	0.9248	4082(116)
HOBr(Br–O)	1.8596	645(14)	HCl(H–Cl)	1.2878	3025(43)
FCN(F–C)	1.2846	1026(63)	HCN(H–C)	1.0779	3456(74)
ClCN(Cl–CN)	1.6502	742(10)	CF_3_H(C–H)	1.0955	3226(21)
BrCN(Br–C)	1.7974	596(2)	CCl_3_H	1.0935	3202(1)
CF_3_Cl(C–Cl)	1.7568	480(1)	N_2_	1.1318	2157(0)
BH_4_^1−^	1.2490	2291(615)	CO(C–O)	1.1502	2072(34)
CH_4_	1.0980	3207(19)			

**Table 3:** Unscaled vibrational frequencies (cm^−1^) with corresponding intensities (values given in parenthesis, km mol^−1^) for complexes

Adduct	*ν*	Δ*ν*	*ν*(B–H)	Δ*ν*(B–H)	*ν*(B–H)	Δ*ν*(B–H)	*ν*(B–H)	Δ*ν*(B–H)	*ν*(B–H)	Δ*ν*(B–H)
BH_4_(H_2_)_f_^1−^	4353(131)(H–H)	−112	2297(556)	6	2297(556)	6	2301(424)	9	2306(201)	15
BH_4_(H_2_)_v_^1−^	4365(126)(H–H)	−100	2297(598)	6	2297(598)	6	2300(330)	8	2313(366)	22
BH_4_(CF_3_Cl)_v_^1−^	485(15)(C–Cl)	5	2309(369)	18	2309(177)	18	2309(465)	17	2333(528)	33
BH_4_(CF_3_CH)_v_^1−^	3202()114(C–H)	−24	2307(449)	16	2307(449)	16	2310(82)	18	2367(480)	67
BH_4_(CO)_v_^1−^	2067(28)(C–O)	−5	2296(515)	5	2296(530)	5	2301(135)	9	2309(500)	9
BH_4_(N_2_)_f_^1−^	2152(1)(N–N)	−5	2299(504)	8	2299(550)	10	2301(61)	9	2306(587)	6
BH_4_(HCN)_f_^1−^	3161(755)(H–C)	−295	2316(429)	25	2316(75)	25	2316(427)	24	2383(486)	83
BH_4_(ClCN)_f_^1−^	732(156)(Cl–N)	−10	2307(446)	16	2307(446)	16	2309(102)	17	2357(510)	57
BH_4_(FCN)_f_^1−^	1054(56)(F–C)	28	2294(562)	3	2294(562)	3	2300(125)	9	2319(493)	28
BH_4_(CH_3_OH)_e_^1−^	3494(682)(O–H)	−349	2280(388)	−11	2328(123)	37	2334(430)	42	2357(507)	57
BH_4_(HCl)_e_^1−^	1388(3875)(H–Cl)	−1611	2228(238)	−46	2383(91)	92	2421(424)	119	2434(323)	137
BH_4_(HF)_e_^1−^	3271(1466)(H–F)	−811	2295(432)	4	2353(164)	62	2373(447)	81	2373(451)	73
BH_4_(HOBr)_v_^1−^	385(652)(O–Br)	−260	1978(5326)	−313	2434(103)	143	2503(289)	211	2511(288)	211
BH_4_(HOCl)_v_^1−^	422(238)(O–Cl)	−318	1932(5488)	−359	2442(124)	151	2522(285)	230	2523(283)	223
BH_4_(H_2_O)_v_^1−^	3543(520)(H–O)	−261	2270(486)	−21	2309(319)	18	2341(298)	49	2351(503)	51
BH_4_(SH_2_)_v_^1−^	2433(657)(S–H)	−321	2256(747)	−35	2320(111)	29	2338(461)	46	2350(492)	50
BH_4_(SHF)_f_^1−^	358(881)(S–F)	−424	1959(3167)	−332	2442(98)	151	2509(247)	217	2532(266)	232
BH_4_(SF_2_)_f_^1−^	466(981)(S–F)	−332	1957(2320)	−334	2430(50)	139	2494(275)	202	2508(293)	208
BH_4_(PH_3_)_f_^1−^	2391(169)(P–H)	−61	2288(436)	−3	2294(339)	3	2305(259)	13	2321(564)	21
BH_4_(PH_2_F)_f_^1−^	540(303)(P–F)	−240	2061(2206)	−230	2386(119)	95	2422(347)	130	2433(282)	133
BH_4_(PHF_2_)_f_^1−^	601(354)(P–F)	−205	2147(1186)	−144	2365(81)	74	2404(384)	112	2423(358)	123
BH_4_(PF_3_)_f_^1−^	648(442)(P–F)	−177	2225(871)	−66	2362(69)	71	2389(355)	97	2399(436)	99
BH_4_(BrCN)_f_^1−^	545(9)(Br–C)	−51	2242(769)	−49	2347(102)	56	2362(411)	70	2373(472)	73
BH_4_(CH_4_)_f_^1−^	3194(12)(C–H)	−13	2297(528)	5	2297(528)	5	2303(248)	3	2311(392)	20
BH_4_(CH_4_)_v_^1−^	3191	−16	2298(583)	6	2298(583)	6	2301(332)	9	2315(416)	15
BH_4_(CCl_3_H)_f_^1−^	3066	−136	2310(345)	18	2310(345)	18	2311(71)	19	2379(523)	79

**Table 4:** Selected bond lengths (Å) of BH_4_(L)^1−^ aggregates at MP2/aug-cc-pVDZ

Adduct	*r*	Δ*r*	*r*(B–H)_1_	Δ*r*(B–H)_1_	*r*(B–H)_2_	Δ*r*(B–H)_2_	*r*(B–H)_3_	Δ*r*(B–H)_3_	*r*(B–H)_4_	Δ*r*(B–H)_4_
BH_4_(H_2_)_f_^1−^	0.7608(H–H)	0.0060	1.2485	−0.0005	1.2486	−0.0005	1.2485	−0.0005	1.2472	−0.0018
BH_4_(H_2_)_v_^1−^	0.7601(H–H)	0.0053	1.24846	−0.0007	1.2484	−0.0007	1.2484	−0.0007	1.2470	−0.0020
BH_4_(CF_3_Cl)_f_^1−^	1.2482(C–Cl)	−0.5086	1.2479	−0.0011	1.2471	−0.0020	1.2471	−0.0020	1.2430	−0.0060
BH_4_(CF_3_CH)_f_^1−^	1.0977(C–H)	0.0022	1.2477	−0.0013	1.2476	−0.0014	1.2477	−0.0014	1.2386	−0.0104
BH_4_(CH_3_OH)_e_^1−^	0.9843(O–H)	0.0186	1.2402	−0.0088	1.2440	−0.0051	1.2440	−0.0051	1.2522	0.0032
BH_4_(CO)_f_^1−^	1.1510(C–O)	0.0008	1.2483	−0.0007	1.2486	−0.0004	1.2486	−0.0005	1.2466	−0.0024
BH_4_(HCl)_e_^1−^	1.4221(H–Cl)	0.3442	1.2350	−0.0140	1.2351	−0.0140	1.2308	−0.0182	1.2704	0.0214
BH_4_(HCN)_f_^1−^	1.1001(H–C)	0.0222	1.2469	−0.0022	1.2469	−0.0022	1.2468	−0.0022	1.2365	−0.0125
BH_4_(HF)_e_^1−^	0.9633(H–F)	0.0385	1.2399	−0.0092	1.2389	−0.0101	1.2391	−0.0100	1.2527	0.0037
BH_4_(HOBr)_v_^1−^	2.0761(O–Br)	0.2165	1.2235	−0.0255	1.2238	−0.0252	1.2254	−0.0236	1.3583	0.1093
BH_4_(HOCl)_v_^1−^	2.0381(O–Cl)	0.3055	1.2227	−0.0264	1.2227	−0.0263	1.2227	−0.0264	1.3956	0.1466
BH_4_(N_2_)_f_^1−^	1.1320(N–N)	0.0002	1.2481	−0.0007	1.2483	−0.0007	1.2482	−0.0008	1.2472	−0.0019
BH_4_(ClCN)_f_^1−^	1.6496(C–Cl)	−0.0006	1.2475	−0.0015	1.2475	−0.0015	1.2475	−0.0015	1.2398	−0.0092
BH_4_(H_2_O)_v_^1−^	0.9832(H–O)	0.0173	1.2469	−0.0021	1.2423	−0.0067	1.2415	−0.0075	1.2533	0.0043
BH_4_(FCN)_f_^1−^	1.2707(F–C)	−0.0139	1.2487	−0.0003	1.2487	−0.0003	1.2487	−0.0003	1.2451	−0.0039
BH_4_(SH_2_)_e_^1−^	1.3778(H–S)	0.0282	1.2452	−0.0038	1.2428	−0.0062	1.2428	−0.0062	1.2536	0.0046
BH_4_(SHF)_f_^1−^	1.9294(S–F)	0.2518	1.2209	−0.0283	1.2261	−0.0229	1.2208	−0.0282	1.3672	0.1182
BH_4_(SF_2_)_f_^1−^	1.8328(S–F)	0.1830	1.2241	−0.0249	1.2267	−0.0223	1.2241	−0.0249	1.3326	0.0836
BH_4_(PH_3_)_f_^1−^	1.4365(P–H)	0.0099	1.2450	−0.0040	1.2474	−0.0016	1.2490	−0.0001	1.2494	0.0004
BH_4_(PH_2_F)_f_^1−^	1.7946(P–F)	0.1219	1.2342	−0.0148	1.2314	−0.0176	1.2331	−0.0160	1.2903	0.0413
BH_4_(PHF_2_)_f_^1−^	1.7458(P–F)	0.0964	1.2385	−0.0106	1.2340	−0.0152	1.2333	−0.0157	1.2748	0.0258
BH_4_(PF_3_)_f_^1−^	1.70768(P–F)	0.0782	1.2361	−0.0130	1.2379	−0.0111	1.2379	−0.0111	1.2635	0.0145
BH_4_(BrCN)_f_^1−^	1.8251(Br–CN)	0.0277	1.2388	−0.0102	1.2407	−0.0083	1.2408	−0.0082	1.25833	0.0093
BH_4_(CH_4_)_f_^1−^	1.0996(C–H)	0.0015	1.2463	0.0027	1.2485	−0.0005	1.2485	−0.0005	1.2485	−0.0005
BH_4_(CH_4_)_v_^1−^	1.0997(C–H)	0.0016	1.2469	−0.0021	1.2481	−0.0009	1.2481	−0.0009	1.2481	−0.0009
BH_4_(CCl_3_H)_v_^1−^	1.1044(C–H)	0.0064	1.2370	−0.0121	1.2473	−0.0017	1.2473	−0.0017	1.2473	−0.0017

In the BH_4_(ClCF_3_)_f_^1−^ complex, a greater proportion of intermolecular interactions could be classified as TrB. A partial charge of +0.0013 resulted from charge transfers from CF_3_Cl to BH_4_^1−^. In addition, some XB interactions appeared between BH_4_^1−^ and ClCF_3_ species. The most intense charge transfers between BH_4_^1−^ and ClCF_3_ returned to lp(Cl) → σ*_(B–H4)_. Due to this charge transfer, we saw the most variations in the bond length and frequencies for bonds directly involved in these interactions. The data given in Tables 2–4 show that B–H stretching frequencies for BH_4_(CF_3_Cl)_f_^1−^ had a blue shift (17–33 cm^−1^) along with 0.0011 to 0.0060 Å reductions in their bond distances. The greatest variations (33 cm^−1^, 0.0060 Å) were seen for B–H *trans* to the CF_3_Cl molecule. In addition, for CF_3_–Cl, a contraction of ∼0.5086 Å and a blue shift of 5 cm^−1^ were obtained.

For BH_4_(CO)_f_^1−^, the interaction of CO as a sigma donor π-acceptor molecule with BH_4_^1−^ elicited some information about the nature of intermolecular contacts. BH_4_^1−^, through its B–H bonds as electron donors, interacted with the CO molecule. In contrast, the CO molecule interacted with the σ holes of BH_4_^1−^ through its π-bonds and lone pairs, particularly the σ hole related to the B–H3 bond of BH_4_^1−^. The sum of these intermolecular interactions directed the CO molecule to align with a triangular face of BH_4_^1−^. The presence of a partial charge of +0.0007 for the CO molecule suggested the preference of TrB in the optimized structure of BH_4_(CO)_f_^1−^.

In BH_4_(N_2_)_f_^1−^, the N_2_ molecule, to some extent, had the characteristics of CO, but it was a weaker σ-donor and had weaker π-acceptor properties than CO. Therefore, its interactions mainly occurred as an electron donor molecule with σ*_(B–H3)_ or σ-hole prolongation to the B–H3 bond. Electron donation by the N_2_ molecule could be provided from lone pairs or N–N bonding electrons. The data given for the NBO (Table 5) show the presence of a 0.0030 partial charge for the N_2_ molecule, which indicates N_2_ → BH_4_ charge transfer. Also, for BH_4_(CO)_f_^1−^ and BH_4_(N_2_)_f_^1−^, we can see B–H bond contraction of 0.0004–0.0024 and 0.007–0.0019 Å along with blue shift for B–H vibrational frequencies of 5–9 and 6–10 cm^−1^. On the other hand, a red shift of 5 cm^−1^ and bond elongation of 0.0008 and 0.0002 Å were seen for CO and N_2_ molecules, respectively.

**Table 5:** NBO charge transfer (*Q*) of the BH_4_(L)^1−^ complexes at the MP2/aug-cc-pVDZ level of theory

Complex	Donor	Acceptor	*E* ^2^	*Q*(L)	Complex	Donor	Acceptor	*E* ^2^	*Q*(L)
BH_4_(ClCF_3_)_f_^1−^	BD_(B–H2)_	BD*_(C–Cl)_	0.23	0.0013	BH_4_(ClCN)_f_^1−^	BD_(B–H2)_	BD*_(C–Cl)_	0.59	−0.0021
	BD_(B–H3)_	BD*_(C–Cl)_	0.27			BD_(B–H3)_	BD*_(C–Cl)_	0.27	
	BD_(B–H4)_	BD*_(C–Cl)_	0.20			BD_(B–H4)_	BD*_(C–Cl)_	0.59	
	BD_(B–H5)_	BD*_(C–Cl)_	0.53			BD_(B–H5)_	BD*_(C–Cl)_	0.59	
	LP_(Cl)_	BD*_(B–H2)_	0.56			LP_(Cl)_	BD*_(B–H2)_	0.69	
	LP_(Cl)_	BD*_(B–H3)_	0.57			LP_(Cl)_	BD*_(B–H3)_	3.5	
	LP_(Cl)_	BD*_(B–H4)_	2.97			LP_(Cl)_	BD*_(B–H4)_	0.69	
	LP_(Cl)_	BD*_(B–H5)_	0.57			LP_(Cl)_	BD*_(B–H5)_	0.69	
BH_4_(FCN)_f_^1−^	LP_(F)_	BD*_(B–H2)_	0.34	0.0015	BH_4_(HCF_3_)_f_^1−^	BD_(B–H2)_	BD*_(C–H10)_	4.00	−0.0151
	LP_(F)_	BD*_(B–H3)_	1.58			BD_(B–H4)_	BD*_(C–H10)_	3.92	
	LP_(F)_	BD*_(B–H4)_	0.34			BD_(B–H5)_	BD*_(C–H10)_	3.96	
	LP_(F)_	BD*_(B–H5)_	0.34						
BH_4_(BrCN)_f_^1−^	BD_(B–H2)_	BD*_(C–Cl)_	9.67	−0.0388		LP_(Br)_	BD*_(B–H2)_	1.10	−0.0388
	BD_(B–H3)_	BD*_(C–Cl)_	0.44			LP_(Br)_	BD*_(B–H3)_	2.91	
	BD_(B–H4)_	BD*_(C–Cl)_	0.13			LP_(Br)_	BD*_(B–H4)_	0.5	
	BD_(B–H5)_	BD*_(C–Cl)_	0.13			LP_(Br)_	BD*_(B–H5)_	0.5	
BH_4_(HCl)_e_^1−^	BD_(B–H4)_	BD*_(H–Cl)_	94.45	−0.1702	BH_4_(HF)_e_^1−^	BD_(B–H4)_	BD*_(H–F)_	22.39	−0.0452
	BD_(B–H5)_	BD*_(H–Cl)_	1.35			BD_(B–H3)_	BD*_(H–F)_	0.41	
	BD_(B–H2)_	BD*_(H–Cl)_	0.35			BD_(B–H2)_	BD*_(H–F)_	2.91	
	BD_(B–H5)_	BD*_(H–Cl)_	0.35						
BH_4_(HCN)_f_^1−^	BD_(B–H2)_	0.25	BD*_(N–C)_	−0.0214	BH_4_(HOCl)_v_^1−^	BD_(B–H3)_	BD*_(H–Cl)_	7.65	−0.5059
	BD_(B–H2)_	4.06	BD*_(C–H)_			BD_(B–H4)_	BD*_(H–Cl)_	7.21	
	BD_(B–H3)_	0.69	BD*_(C–H)_			BD_(B–H5)_	BD*_(H–Cl)_	7.44	
	BD_(B–H4)_	0.17	BD*_(N–C)_			BD_(H–Cl)_	σ*_(B)_		
BH_4_(HOBr)_v_^1−^	BD_(B–H3)_	σ*_(Br)_	213.11	−0.3757	BH_4_(N_2_)_f_^1−^	BD_(B–H2)_	BD*_(N–N)_	0.11	0.0030
	LP_(BR)_	BD*_(B–H3)_	13.40			BD_(N–N)_	BD*_(B–H3)_	0.43	
						LP_(N6)_	BD*_(B–H3)_	0.39	
						LP_(N7)_	BD*_(B–H3)_	0.34	
BH_4_(CH_3_OH)_v_^1−^	BD_(B–H5)_	BD*_(O–H)_	11.23	−0.0188	BH_4_(H_2_O)_v_^1−^	BD_(B–H5)_	BD*_(O–H)_	8.13	−0.0138
	BD_(B–H4)_	BD*_(O–H)_	0.49						
	BD_(B–H3)_	BD*_(O–H)_	0.27						
	BD_(B–H2)_	BD*_(O–H)_	0.27						
BH_4_(SFH)_v_^1−^	BD_(B–H2)_	BD*_(S–H)_	2.26	−0.4264	BH_4_(SF_2_)_v_^1−^	BD_(B–H2)_	BD*_(F–S)_	1.51	−0.3847
	BD_(B–H3)_	BD*_(S–H)_	9.62			BD_(B–H3)_	LP_(S)_	0.78	
	BD_(B–H4)_	BD*_(S–H)_	4.30			BD_(B–H4)_	LP_(S)_	0.99	
	BD_(B–H5)_	BD*_(S–F)_	286.14			BD_(B–H5)_	BD*_(S)_	220.27	
	BD_(S–H)_	BD*_(B–H2)_	1.61			BD_(B–H5)_	BD*_(F–S)_	9.46	
	BD_(S–H)_	BD*_(B–H3)_	2.66			LP_(S)_	BD*_(B–H2)_	0.53	
	BD_(S–H)_	BD*_(B–H4)_	1.67			LP_(S)_	BD*_(B–H3)_	0.62	
	LP_(S)_	BD*_(B–H2)_	1.03			LP_(S)_	BD*_(B–H4)_	0.63	
	LP_(S)_	BD*_(B–H3)_	0.22			LP_(S)_	BD*_(B–H5)_	3.10	
	LP_(S)_	BD*_(B–H4)_	1.23			LP_(S)_	BD*_(B–H3)_	1.20	
	LP_(S)_	BD*_(B–H5)_	0.45			LP_(S)_	BD*_(B–H4)_	1.20	
	LP_(S)_	BD*_(B–H3)_	0.76						
BH_4_(SH_2_)_v_^1−^	BD_(B–H2)_	BD*_(S–H)_	0.06	−0.0295	BH_4_(CO)_f_^1−^	BD_(B–H4)_	BD*_(C–O)_	0.23	0.0007
	BD_(B–H3)_	BD*_(S–H)_	0.79			BD_(B–H5)_	BD*_(C–O)_	0.24	
	BD_(B–H4)_	BD*_(S–H)_	0.79			BD_(C–O)_	BD*_(B–H3)_	0.45	
	BD_(B–H5)_	BD*_(S–H)_	13.14			LP_(C)_	BD*_(B–H3)_	0.72	
	BD_(S–H8)_	BD*_(B–H2)_	0.07			LP_(C)_	BD*_(B–H4)_	0.18	
	BD_(S–H8)_	BD*_(B–H3)_	0.65			LP_(C)_	BD*_(B–H5)_	0.18	
	BD_(S–H8)_	BD*_(B–H4)_	0.22			LP_(O)_	BD*_(B–H3)_	0.17	
	LP_(S)_	BD*_(B–H5)_	0.08						
	LP_(S)_	BD*_(B–H3)_	0.07						
	LP_(S)_	BD*_(B–H5)_	0.06						
	LP_(S)_	BD*_(B–H2)_	0.15						
BH_4_(PH_3_)_v_^1−^	BD_(B–H2)_	BD*_(P–H8)_	0.12	−0.0039	BH_4_(PH_2_F)_v_^1−^	BD_(B–H2)_	BD*_(P–F7)_	0.46	−0.1773
	BD_(B–H4)_	BD*_(P–H8)_	0.61			BD_(B–H5)_	BD*_(P–F7)_	47.07	
	BD_(B–H5)_	BD*_(P–H7)_	0.12			BD_(B–H5)_	BD*_(P–H8)_	4.83	
	BD_(B–H5)_	BD*_(P–H9)_	0.84			BD_(B–H5)_	BD*_(P–H9)_	4.96	
	BD_(P–H7)_	BD*_(B–H2)_	0.57			BD_(P–H8)_	BD*_(B–H2)_	1.06	
	BD_(P–H8)_	BD*_(B–H4)_	0.12			BD_(P–H9)_	BD*_(B–H5)_	0.64	
	BD_(P–H8)_	BD*_(B–H4)_	0.12			lp(P)	BD*_(B–H5)_	9.09	
BH_4_(PHF_2_)_v_^1−^	BD_(B–H5)_	BD*_(P–H7)_	7.94	−0.1469	BH_4_(PF_3_)_v_^1−^	BD_(B–H5)_	BD*_(P–F7)_	5.24	−0.0974
	BD_(B–H5)_	BD*_(P–H8)_	29.10			BD_(B–H5)_	BD*_(P–F8)_	18.37	
	BD_(B–H5)_	BD*_(P–H9)_	3.49			BD_(B–H5)_	BD*_(P–H9)_	5.25	
	BD_(P–H9)_	BD*_(B–H2)_	1.59			lp(P)	BD*_(B–H4)_	6.38	
	lp(P)	BD*_(B–H4)_	1.27						
	lp(P)	BD*_(B–H5)_	1.24						
BH_4_(H_2_)_f_^1−^	BD_(B–H2)_	BD*_(H–H)_	0.37	−0.0013	BH_4_(H_2_)_v_^1−^	BD_(B–H4)_	BD*_(H–H)_	1.83	−0.0034
	BD_(B–H4)_	BD*_(H–H)_	0.37			BD_(H–H)_	BD*_(B–H4)_	0.12	
	BD_(B–H5)_	BD*_(H–H)_	0.37						
BH_4_(CH_4_)_f_^1−^	BD_(B–H2)_	BD*_(C–H7)_	0.36	−0.0027	BH_4_(CCl_3_H)_v_^1−^	BD_(H1–B)_	BD*_(C–H)_	6.49	−0.0244
	BD_(B–H3)_	BD*_(C–H7)_	0.30			BD_(H1–B)_	BD*_(C–Cl8)_	0.26	
	BD_(B–H4)_	BD*_(C–H7)_	0.36			BD_(H2–B)_	BD*_(C–H)_	6.47	
	BD_(B–H5)_	BD*_(C–H7)_	0.36 0.06			BD_(H2–B)_	BD*_(C–Cl9)_	0.26	
	BD_(C–H7)_	BD*_(B–H2)_	0.38			BD_(H3–B)_	BD*_(C–H)_	6.56	
	BD_(C–H7)_	BD*_(B–H3)_	0.06			BD_(H3–B)_	BD*_(C–Cl7)_	0.27	
	BD_(C–H7)_	BD*_(B–H4)_	0.06			BD_(H4–B)_	BD*_(C–H)_	0.63	
	BD_(C–H7)_	BD*_(B–H5)_							
BH_4_(CH_4_)_v_^1−^	BD_(H1–B)_	BD*_(C–H9)_	1.59	−0.0025					
	BD_(H2–B)_	BD*_(C–H9)_	0.11						
	BD_(H3–B)_	BD*_(C–H9)_	0.11						
	BD_(H4–B)_	BD*_(C–H9)_	0.11						

For the BH_4_(BrCN)_f_^1−^ adduct, in addition to XB, we could have BrCN → BH_4_ charge transfer (TrB) as part of the interaction between fragments. Hence, in this case, a net partial charge of −0.0388 for BrCN could be considered to be a result of an XB interaction. Comparison of interaction energies obtained by NBO (Table 5) analyses indicated that the contribution of the halogen bond was more significant than that of the triel bond between BrCN and BH_4_^1−^. In BH_4_(BrCN)_f_^1−^, we had a 49 cm^−1^ red shift and 0.0093 Å elongation for B–H *trans* to the BrCN molecule, and a blue shift of 56–73 cm^−1^ besides a 0.0082-to-0.0102 Å reduction for other B–H bond distances. In contrast, for the Br–C distance of the BrCN molecule, a bond elongation of 0.0277 and red shift of 51 cm^−1^ were seen.

For the BH_4_(ClCN)_f_^1−^ aggregate, in addition to the L → BH_4_ charge transfer (TrB), XB as another important interaction between BH_4_^1−^ and ClCN could be possible. Hence, in this case, the net partial charge −0.0021 of ClCN could be considered to be a result of an XB interaction. Comparison of the interaction energies obtained by NBO analysis indicated that the contribution of the triel bond was a significant part of interactions between ClCN and BH_4_^−1^ and it provided a stronger stabilization effect compared with the halogen bond. In BH_4_(ClCN)_f_^1−^, we had a 16–57 cm^−1^ blue shift besides a 0.0015-to-0.0092 Å reduction in B–H bond distances. The greatest changes (57 cm^−1^, 0.0092 Å) were observed in the B–H *trans* to the ClCN molecule. In contrast, in the ClCN molecule, a bond contraction of 0.0006 and a red shift of 10 cm^−1^ were observed for the Cl–CN bond.

As a result, the nature of the intermolecular interactions had a crucial role in determining the stability and properties of the BH_4_(L)_v_^1−^ adducts. Therefore, as a common principle, the triel bond played a significant part in shaping adducts between BH_4_^−1^ and N_2_, CO, FCN, ClCN, and ClCF_3_ molecules.

In these adducts, for TrB intermolecular interactions, L must be in the appropriate orientation to a σ hole of BH_4_^1−^. Hence, it can be seen from the optimized structures of BH_f_(FCN)_v_^1−^, BH_4_(ClCN)_f_^1−^, BH_4_(BrCN)_f_^1−^, BH_4_(ClCF_3_)_f_^1−^, BH_4_(N_2_)_f_^1−^ and BH_4_(CO)_f_^1−^ aggregates that the L molecule is often coaxing with a σ hole of BH_4_^1−^. In other words, the preferred location for L is situated on a triangular face of the BH_4_^1−^ anion.

### XB complexes BH_4_(L)_v_^1−^

The tetrahidroborate anion formed halogen-bonded BH_4_(ClOH)_v_^1−^ and BH_4_(BrOH)_v_^1−^ aggregates with HOCl and HOBr molecules (Fig. 2). The stability of these adducts was −18.69 and −23.76 kcal mol^−1^, respectively, which were more stable than the previously discussed complexes. Partial charges of −0.5059 for HOCl and −0.3757 for HOBr suggested more charge transfers from BH_4_^1−^ to L for these adducts. For BH_4_(ClOH)_v_^1−^ and BH_4_(BrOH)_v_^1−^, for a strong halogen bond, one hydride atom moved away from BH_4_^1−^ and approached the halogen atom of hypohalid acid. Therefore, because of the strong halogen bond interaction, HOBr and HOCl preferred to interact with a vertex B–H rather than the triangular faces of BH_4_^1−^. Thus, more stable halogen bond formation might be the primary driving force for the directionality and structural preference for these adducts.

For the XB complexes BH_4_(HOCl)_v_^1−^ and BH_4_(HOBr)_v_^1−^, elongations of 0.3055 and 0.2165 Å along with red shifts of 318 and 260 cm^−1^ were observed for O–X bonds. For the BH_4_^1−^ moiety, elongations of 0.1466 and 0.1093 Å and red shifts of 359 and 313 cm^−1^ for B–H involved in the interaction were observed, along with a bond contraction of 0.0264 and 0.0236–0.0255 Å and blue shifts of 151, 223, 230 and 143, 211 cm^−1^ were observed for other B–H bonds when X = Cl and Br, respectively.

### DHB aggregates

BH_4_(HCl)_e_^1−^, BH_4_(HF)_e_^1−^, BH_4_(HCN)_f_^1−^, BH_4_(HCF_3_)_f_^1−^, BH_4_(HCCl_3_)_f_^1−^, BH_4_(HOCH_3_)_v_^1−^, BH_4_(H_2_O)_v_^1−^, BH_4_(H_2_)_f_^1−^, BH_4_(H_2_)_v_^1−^, BH_4_(CH_4_)_f_^1−^, BH_4_(CH_4_)_v_^1−^ and BH_4_(H_2_S)_v_^1−^ were the next categories of aggregates optimized as dihydrogen-bonded adducts (Fig. 2). It seems that the driving force behind the formation of these complexes was the dihydrogen bond interaction between BH_4_^1−^ (dihydrogen bond acceptor) and the counterpart molecule (dihydrogen bond donor). The data in Tables 1 and S2 show that the stabilities of these adducts were in the order:BH_4_(HCN)_f_^1−^ > BH_4_(HCl)_e_^1−^ > BH_4_(HF)_e_^1−^ > BH_4_(HCCl_3_)_v_^1−^ > BH_4_(HCF_3_)_v_^1−^ > BH_4_(HOCH_3_)_v_^1−^ > BH_4_(H_2_O)_v_^1−^ > BH_4_(H_2_S)_v_^1−^ > BH_4_(CH_4_)_f_^1−^ > BH_4_(CH_4_)_v_^1−^ > BH_4_(H_2_)_f_^1−^ > BH_4_(H_2_)_v_^1−^

Interaction of CH_3_OH, H_2_O, or H_2_S molecules with BH_4_^1−^ gave simple dihydrogen bond complexes in which the former molecules acted as dihydrogen bond donors and BH_4_^1−^ acted as a dihydrogen bond acceptor. The results of these interactions were BH_4_(HOCH_3_)_v_^1−^, BH_4_(H_2_O)_v_^1−^ and BH_4_(H_2_S)_v_^1−^ dihydrogen bonded adducts, respectively. These structures showed distortion from the vertex towards edge interactions, and L was very close to a B–H apex with respect to the other one. Therefore, we classified them as BH_4_(L)_v_^1−^ aggregates. In the case of BH_4_(CH_3_OH)_v_^1−^, the O–H bonds exhibited an elongation of 0.0186 Å and a red shift of 349 cm^−1^. On the other hand, 0.0032 Å lengthening and 11 red shifts for B–H involved in DHB (B–H⋯H–O), but 0.0051-to-0.0088 contractions and blue shifts of 37, 42, 57 cm^−1^ for other B–H bonds were observed.

For the DHB adduct BH_4_(H_2_O)_v_^1−^, 0.0173 Å elongations along with a red shift of 261 cm^−1^ for the H–O bond involved in DHB were observed. For the BH_4_^1−^ moiety, an elongation of 0.0043 Å and red shift of 21 cm^−1^ for B–H in DHB, and bond contractions of 0.0021, 0.0067, and 0.0075 Å and blue shifts of 92, 119, and 137 cm^−1^ for the remainder of the B–H bonds were observed.

In the DHB complex BH_4_(H_2_S)_v_^1−^, an elongation of 0.0282 Å along with a red shift of 321 cm^−1^ for the H–S bond involved in DHB was observed. For the BH_4_^1−^ moiety, an elongation of 0.0046 Å and red shift of 35 cm^−1^ for the B–H encountered in DHB, and bond contractions of 0.0038 and 0.0062 Å and blue shifts of 29, 46, and 50 cm^−1^ for the remainder of the B–H bonds were observed.

HCl and HF, as dihydrogen bond donors, formed the DHB complexes BH_4_(HCl)_e_^1−^ and BH_4_(HF)_e_^1−^, respectively, with BH_4_^1−^ as a dihydrogen bond acceptor. As seen from Fig. 2, in these adducts, HCl and HF chose an unsymmetrical bifurcated dihydrogen bond interaction with BH_4_^1−^. Hence, the preferred direction for these molecules was an unsymmetrical bifurcated dihydrogen bond interaction in which, along an edge, they interacted with BH_4_^1−^.

In these adducts, elongations of 0.3442 and 0.0385 Å along with red shifts of 1611 and 811 cm^−1^ were observed for the H–X bonds in BH_4_(HCl)_e_^1−^ and BH_4_(HF)_e_^1−^ complexes. For the BH_4_^1−^ moiety, elongation of 0.0214 and 0.0037 Å, a red shift of 46 cm^−1^ and blue shift of 4 cm^−1^ for B–H involved in the DHB interaction were observed. Also, for the remaining B–H bonds, bond contraction of 0.0140, 0.0182 and 0.0101, 0.0100, and 0.0092 Å and blue shifts of 92, 119, 137, and 62, 73, and 81 cm^−1^ were observed when X = Cl and F, respectively.

In addition to DHB, another type of intermolecular interaction helped to increase the stabilization of some of these adducts. For example, in BH_4_(HCN)_f_^1−^ with stabilization energy of −18.28, in addition to DHB interaction, HCN can have TtB with BH_4_^1−^, a type of noncovalent bond in which a group-14 element (C, Si, Ge, Sn, Pb) as a Lewis acid interacts with a Lewis base. The TtB leads to greater stability of BH_4_(HCN)_f_^1−^ relative to BH_4_(HCl)^1−^ and BH_4_(HF)^1−^ aggregates. Therefore, these two interactions make it more stable than the other ones. In both interactions, BH_4_^1−^ has the role of electron donor and HCN is the electron acceptor. The B–H bonds of BH_4_(HCN)_f_^1−^ showed a 5–83 cm^−1^ blue shift of their vibrational frequency and a 0.0022–0.0125 Å decrease in their bond lengths with adduct formation. Most blue shifts (83 cm^−1^) and bond contraction (0.0022) were seen for the B–H bond, which was *trans* to the intermolecular interaction. These intermolecular interactions reduced the σ(B–H) to σ*(B–H) charge transfers, resulting in stronger B–H bonds. In the case of the HCN molecule, a 0.0222 Å increase in the H–C bond distance and red shift of 295 cm^−1^ were found with adduct formation.

In BH_4_(HCF_3_)_v_^1−^, HCF_3_, as a hydrogen bond donor, stayed coaxial with BH_4_^1−^, and most of the charge transfers occurred for σ_(B–H)_ to the σ*_(H–CF3)_ orbitals in a trifurcated dihydrogen bond interaction. With a stabilization energy of −14.55, it had moderate stability between the studied adducts. For the BH_4_(HCF_3_)_v_^1−^ adduct, a blue shift of 67 cm^−1^ with a 0.0104 Å decrease in the B–H bond *trans* to HCF_3_, as well as a contraction of 16 and 18 Å with a blue shift of 16 and 18 cm^−1^ for other B–H bonds, was found. Conversely, in the CF_3_H molecule, for the C–H bond, a red shift of 24 cm^−1^ and bond elongation of 0.0022 Å were obtained.

In BH_4_(CHCl_3_)_v_^1−^, the CHCl_3_ (as a HBD) remained on the face of the BH_4_^1−^ (as a DHA), and by trifurcated DHB interacted with the tetrahydroborate anion. Charge transfers occurred from σ_(B–H)_ orbitals to σ*_(H–CCl3)_. A stabilization energy of −17.05 kcal mol^−1^ indicated relatively strong DHB in this aggregate. For the BH_4_(HCCl_3_)_v_^1−^ adduct, a 79 cm^−1^ blue shift along with a 0.0121 Å decrease for B–H bond prolongation to HCCl_3_, as well as a 17 Å contraction with 18 and 19 cm^−1^ blue shifts for other B–H bonds, were observed. On the other hand, for the C–H bond of the CCl_3_H molecule, a red shift of 136 cm^−1^ and bond elongation of 0.0110 Å were obtained.

In contrast to the SH_2_ formed by DHB, the interaction between HSF and SF_2_ with BH_4_^1−^ could be considered to be a combination of ChB and TrB that resulted in BH_4_(HSF)_v_^1−^ and BH_4_(SF_2_)_v_^1−^ complexes. The stabilization energies of these aggregates were −26.57 and −24.28 kcal mol^−1^, so they were more stable than other studied systems. More significant interactions in these adducts were seen for σ_(B–H5)_ as a ChB acceptor with σ*_(S–F)_ as a ChB donor. In addition, the contribution of ChB increased from BH_4_(HSF)_v_^1−^ to BH_4_(SF_2_)_v_^1−^, and comparison of their structures showed a greater vertex characteristic in BH_4_(HSF)_v_^1−^ with respect to BH_4_(SF_2_)_v_^1−^. A more detailed analysis of NBO data indicated some additional σ_(B–H)_ to σ*_(S–H)_ charge transfers in BH_4_(HSF)_v_^1−^, which led to greater stability of this adduct with respect to the BH_4_(SF_2_)_v_^1−^ complex.

For the ChB adducts BH_4_(SHF)_v_^1−^ and BH_4_(SF_2_)_v_^1−^, elongations of 0.2518 and 0.1830 Å along with red shifts of 424 and 332 cm^−1^ were observed for S–F bonds *trans* to S⋯H interactions. For the BH_4_^1−^ moiety, elongations of 0.1182 and 0.0836 Å and red shifts of 332 and 334 cm^−1^ for B–H involved in B–H⋯S interactions were noted; bond contractions of 0.0229, 0.0282, 0.0283 and 0.0223, and 0.0249 Å and blue shifts of 151, 217, 232 and 139, 202, and 208 cm^−1^ for other B–H bonds, were observed for BH_4_(SHF)_v_^1−^ and BH_4_(SF_2_)_v_^1−^, respectively.

To investigate the interplay between the PnB and TrB, interactions of BH_4_^1−^ with PH_3_, PH_2_F, PHF_2_, and PF_3_ molecules were considered. The stabilities of related adducts were in the order:BH_4_(PH_2_F)_v_^1−^ > BH_4_(PHF_2_)_v_^1−^ > BH_4_(PF_3_)_v_^1−^ > BH_4_(PH_3_)_v_^1−^

A combination of weak DHB and PnB interactions led to a BH_4_(PH_3_)_v_^1−^ adduct with a stabilization energy of −4.41 kcal mol^−1^. The nature of interactions moved to PnB in more fluorinated phosphines. In BH_4_(PH_3_)_v_^1−^, a PnB interaction appeared between σ_(B–H5)_ as an electron donor and σ*_(P–H9)_ as an electron acceptor; simultaneously, a DHB interaction occurred between σ_(B–H4)_ as an electron donor and σ*_(P–H8)_ as an electron acceptor. The presence of both interactions in this adduct required a specific orientation of components to cause effective contact between overlapping orbitals. Also, a partial charge of −0.0039 indicated charge transfers from BH_4_^1−^ to PH_3_.

BH_4_(PH_2_F)_v_^1−^ contained a PnB interaction between σ_(B–H5)_ as an electron donor and σ*_(P–F7)_ and σ hole P–F7, as an electron acceptor, as well as TrB by LP(P) to σ*_(B–H5)_ charge transfers. BH_4_(PH_2_F)_v_^1−^, with SE of −18.36 kcal mol^−1^, was the most stable complex in this series. The BHP bond angle of 143° enhanced weak interactions, contributing to adduct formation and greater stabilization of the corresponding aggregate.

BH_4_(PHF_2_)_v_^1−^ was mainly obtained through a PnB interaction between σ_(B–H5)_ as an electron donor and the σ hole of P–F8, σ*_(P–F8)_ as an electron acceptor, besides a weak TrB resulting from LP(P) to σ*_(B–H)_ interactions. BH_4_(PHF_2_)_v_^1−^, with SE of −17.57 kcal mol^−1^ and BHP bond angle of 125°, was in the next level of stability from phosphine adducts. This angle was reached due to weak TrB interactions that included adduct formation, and made the complex more stable.

Similarly, BH_4_(PF_3_)_v_^1−^ was primarily obtained by a PnB interaction between σ_(B–H5)_ as an electron donor and σ*_(P–F8)_ as an electron acceptor. Also, a weak TrB was found for LP(P) to σ*_(B–H4)_ and other σ*_(B–H)_ orbitals charge transfers. BH_4_(PF_3_)_v_^1−^, with SE of −14.81 kcal mol^−1^ and BHP angle 117°, was in the third order of stability between phosphine complexes. This angle was observed due to some weaker interactions that helped the greater stabilization of complexes. The HPF and HPH bond angles were 167, 167, 170, and 149° for BH_4_(PF_3_)_v_^1−^, BH_4_(PHF_2_)_v_^1−^, BH_4_(PH_2_F)_v_^1−^, BH_4_(PH_3_)_v_^1−^, respectively. These data indicated that, especially in the case of fluorinated phosphines, this angle was less affected by a change in the number of F atoms on the phosphine molecule. On the other hand, results for fluorinated phosphines indicated that increasing the number of F atoms in phosphine molecules led to banishment of TrB and, therefore, a reduction in the stability of the related adducts.

For the PnB adducts BH_4_(PH_3_)_v_^1−^, BH_4_(PH_2_F)_v_^1−^, BH_4_(PHF_2_)_v_^1−^ and BH_4_(PF_3_)_v_^1−^, elongations of 0.0099, 0.1219, 0.0964 and 0.0782 Å along with red shifts of 61, 240, 205, and 177 cm^−1^ were observed for P–H and P–F bonds *trans* to P⋯H interactions. For the BH_4_^1−^ moiety, elongations of 0.0004, 0.0413, 0.0258 and 0.0145 Å and red shifts of 3, 230, 144, and 66 cm^−1^ for B–H *trans* to the B–H⋯P interaction were observed. Also, bond contractions of 0.0001, 0.0016, 0.0040 and 0.0106, 0.0152, 0.0157 and 0.0111, 0.0130 Å, as well as blue shifts of 3, 13, 21 and 95, 130, 133 and 74, 112, 123, and 71, 97, 99 cm^−1^, were observed for other B–H bonds of adducts, respectively.

From the interaction of BH_4_^1−^ with the H_2_ molecule, BH_4_(H_2_)_f_^1−^ and BH_4_(H_2_)_v_^1−^, as local minima adducts, and BH_4_(H_2_)_e_^1−^, as an nonlocal structure, were optimized. The stabilities of these adducts were −0.57, −0.01 and −0.39 kcal mol^−1^, respectively. Hence, face-centered interactions aided adduct formation relative to head-to-head counterparts. The partial charge of components indicated that, in both adducts, the electron acceptor ability of H_2_ was preferred. In BH_4_(H_2_)_f_^1−^, trifurcated DHB and TrB could be seen between interacting components. However, for BH_4_(H_2_)_v_^1−^, an orbital overlap between σ_(B–H4)_ as an electron donor and σ*(H–H) as an electron acceptor led to a conventional dihydrogen-bonded adduct. In comparison, H_2_ molecules preferred to interact through a triangular face rather than a vertex or edge interaction.

Interaction of BH_4_^1−^ with the CH_4_ molecule led to BH_4_(CH_4_)_f_^1−^ and BH_4_(CH_4_)_v_^1−^ as local minima and BH_4_(CH_4_)_e_^1−^ as a nonlocal structure, which stabilities of −2.35, −1.52 and −2.07 kcal mol^−1^, respectively. Results show that facial adduct is more stable than the adduct that formed by vertex-to-vertex interaction. The partial charge of components indicates that in both adducts, the electron acceptor ability of CH_4_ is preferred. In the BH_4_(H_2_)_f_^1−^, a trifurcated DHB a TrB bond can be seen between interacting components. But for BH_4_(H_2_)_v_^1−^ an orbital overlap between σ_(B–H4)_ as an electron donor and σ*(H–H) as an electron acceptor leads to a conventional dihydrogen bond adduct. In comparison, CH_4_ molecules prefer to interact through a triangular face rather than a vertex or edge interaction.

The vibrational stretching frequencies of B–H bonds (Table 2) in free BH_4_^1−^ appeared at 2291 cm^−1^. The results given in Table 3 show that interactions between BH_4_^1−^ and counterpart H_2_ molecules resulted in a blue shift of B–H stretching vibrations. Hence, the B–H stretching vibrations in BH_4_(H_2_)^1−^ aggregates led to a 6–22 cm^−1^ blue shift due to adduct formation. Moreover, the blue shift in BH_4_(H_2_)_v_^1−^ (22 cm^−1^) belonged to a B–H bond that interacted directly with the H_2_ molecule. Also, for BH_4_(H_2_)_f_^1−^, a 15 cm^−1^ blue shift belonged to a B–H bond in the *trans* direction relative to the interaction center. These blue shifts led to a 0.0005-to-0.0020 Å decrease in B–H bond lengths (Table 4). Most contractions returned to those B–H bonds that showed the greatest blue shift in their stretching frequencies. For example, a 0.0020 Å decrease was observed for the B–H bond involved in the interaction for BH_4_(H_2_)_v_^1−^ and, similarly, 0.0018 Å was ascribed to the B–H in the *trans* position relative to the center of the interaction. In contrast, for H_2_ molecules, we observed red shifts of −100 and −112 cm^−1^ in BH_4_(H_2_)_v_^1−^ and BH_4_(H_2_)_f_^1−^ aggregates, respectively. These red shifts were in agreement with the 0.0053 and 0.0060 Å increases in the H_2_ bond distances for BH_4_(H_2_)_v_^1−^ and BH_4_(H_2_)_f_^1−^ aggregates. These changes occurred due to the BH_4_^1−^ to H_2_ charge transfers that led to the strengthening of B–H bonds and weakening of H_2_ bonds.

### Atoms in molecules (AIM) analysis

**Fig. 3:**
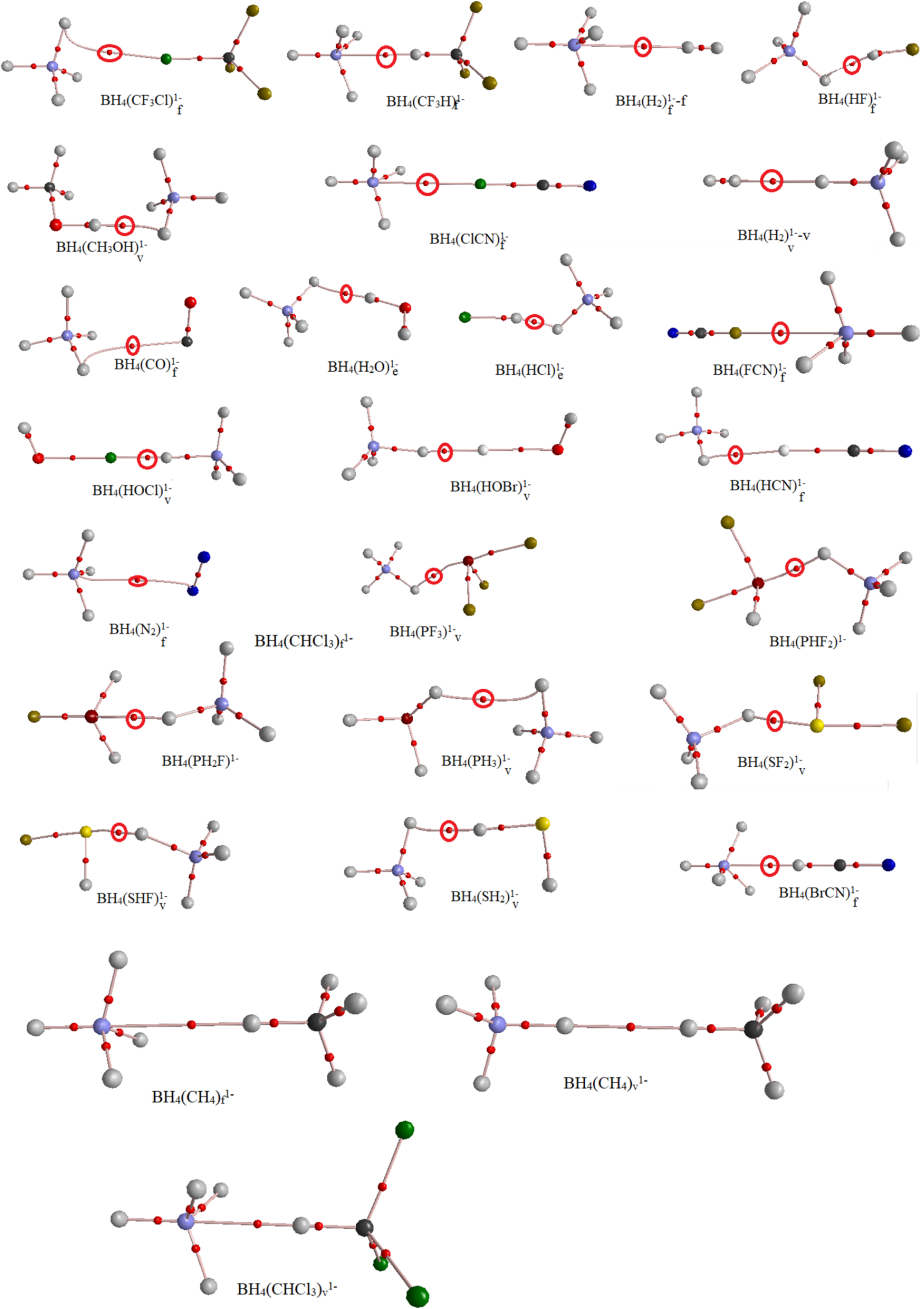
Molecular graphs of BH_4_(L)^1−^ complexes at the MP2/aug-cc-pVDZ level.

The AIM theory^35,36^ was used to study the nature of BH_4_(L)^1−^ interactions. Table 6 and Fig. 3 show the results and molecular graphs of AIM calculations, in which *p* is electron density at intermolecular bond critical points (BCP), *∇*^2^ is the Laplacian, and the −*G*/*V* is the ratio between the kinetic and potential electron energy density at BCP in BH_4_(L)^1−^ complexes. If the gravitational *G* overshadows the potential *V*, then the positive profile of *∇*^2^ indicates a reduction in charge density along the intermolecular bond path. In this case, the bond is known as a “closed-shell interaction”, such as hydrogen bonds or other intermolecular weak bonds.

**Table 6:** Topological parameters for fully optimized BH_4_(L)^1−^ adducts

	*p*	Δ*p*	*G*	*V*	−*G*/*V*	*H*
BH_4_(H_2_)_f_^1−^	0.0055	−0.0045	−0.0037	0.0009	4.2064	−0.0028
BH_4_(H_2_)_v_^1−^	0.0068	−0.0044	0.0039	−0.0005	7.7423	0.0034
BH_4_(CF_3_Cl)_f_^1−^	0.0093	−0.0086	0.0069	−0.0018	3.8655	0.0051
BH_4_(CF_3_H)_f_^1−^	0.0168	−0.0124	0.0119	−0.0004	27.4113	0.0115
BH_4_(CH_3_OH)_v_^1−^	0.0246	−0.0149	0.0147	−0.0002	97.6208	0.0146
BH_4_(CO)_e_^1−^	0.0056	−0.0049	0.0036	−0.0013	2.6724	0.0022
BH_4_(HCl)_e_^1−^	0.0748	−0.0008	0.0303	0.0297	1.0259	0.0660
BH_4_(HCN)_f_^1−^	0.0179	−0.0129	0.0123	−0.0006	21.1163	0.0117
BH_4_(HF)_e_^1−^	0.0357	−0.0189	0.0219	0.0031	7.1625	0.0250
BH_4_(HOBr)_v_^1−^	0.0963	−0.0054	0.0465	0.0411	1.1309	0.0876
BH_4_(HOCl)_v_^1−^	0.1213	0.0087	0.0554	0.0641	0.8643	−0.0087
BH_4_(N_2_)_f_^1−^	0.0044	−0.0039	0.0031	−0.0008	3.9787	0.0023
BH_4_(ClCN)_f_^1−^	0.0110	−0.0102	0.0085	−0.0018	4.7774	0.0067
BH_4_(H_2_O)_v_^1−^	0.0224	−0.0137	0.0133	−0.0004	33.9675	0.0129
BH_4_(FCN)_f_^1−^	0.0062	−0.0077	0.0062	−0.0015	4.2360	0.0047
BH_4_(SH_2_)_v_^1−^	0.0248	−0.0125	0.0135	0.0010	13.1511	0.0145
BH_4_(SHF)_v_^1−^	0.1166	0.0190	0.0501	0.0691	0.7255	0.1191
BH_4_(SF_2_)_v_^1−^	0.1072	0.01286	0.0438	0.0567	0.7732	0.1007
BH_4_(PH_3_)_v_^1−^	0.0077	−0.0064	0.0051	−0.0013	3.9258	0.0038
BH_4_(PH_2_F)_v_^1−^	0.0633	−0.0005	0.0273	0.0268	1.0187	0.0541
BH_4_(PHF_2_)_v_^1−^	0.0538	−0.0045	0.0229	0.0184	1.2449	0.0414
BH_4_(PF_3_)_v_^1−^	0.0454	−0.0075	0.0192	0.011702	1.6454	0.0310
BH_4_(BrCN)_f_^1−^	0.0253	−0.0160	0.0161	0.0001	149.0463	0.0162
BH_4_(CH_4_)_f_^1−^	0.0072	−0.0060	0.0050	−0.00095	5.26316	0.0040
BH_4_(CH_4_)_v_^1−^	0.0082	−0.0053	0.0048	−0.0004	11.1081	0.0044
BH_4_(CCl_3_H)_v_^1−^	0.0227	−0.0152	0.0157	0.0004	35.7	0.0161

The positive values of *∇*^2^ in Table 4 indicate that all interactions in BH_4_(L)^1−^ complexes were closed-shell. In addition, −*G*/*V* > 1 indicated the non-covalent character of these interactions.

### NBO analysis

NBO^37^ calculations were done on BH_4_(L)^1−^ complexes, and showed that these complexes were the products of orbital overlaps between BH_4_^1−^ and L molecules. In the case of boron tetrahydride, several positions might act as electron donors simultaneously, it could also act as an electron acceptor through the σ-holes on its B–H bonds. Table 5 lists the quantity of charges transferred from the donor to the acceptor (*Q*_ct_) for BH_4_(L)^1−^ adducts. According to data given in Table 5, the *Q*_ct_ for L molecules was negative, which indicated that the electron donation of BH_4_^1−^ was preferred to its electron acceptor properties in BH_4_(L)^1−^ adducts.

### Non-covalent interactions (NCI) analysis

NCIs within and between molecules are important in all branches of chemistry. The NCI method provides valuable results to deepen insights about the NCIs present in molecular adducts.^38,39^ The NCI method visualizes noncovalent interactions, including hydrogen bonding (attractive interactions), steric repulsions, and van der Waals forces within structures involving NCIs. Therefore, to distinguish the repulsive van der Waals interactions and electrostatic forces present in the BH_4_(L)^1−^ adducts, NCI calculations were conducted.

**Fig. 4:**
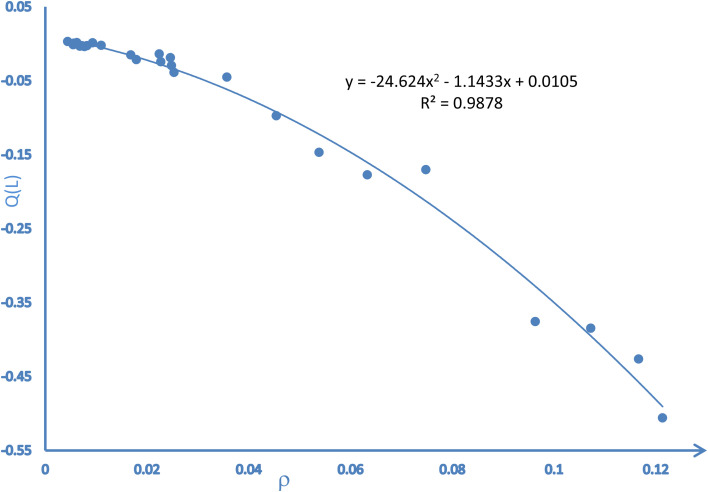
Correlation between *Q*(L) (transferred charges, NBO) and *ρ* (electron density, AIM) for BH_4_(L)^1−^ adducts.

The findings from the NCI analysis are presented in 2D RDG plots and 3D topological representations. We determined the types of interactions happening in the system using the NCI reduced density gradient approach.^38,39^Fig. 4 and S1 are NCI scatter plots, which represent the relationship between the sign of the second Hessian eigenvalue (sign*λ*_2_*ρ*) and RDG. This plot indicated that weak attractive interactions were present between BH_4_^1−^ and L within BH_4_(L)^1−^ adducts.

The parameter sign(*λ*_2_) *ρ* > 0 illustrates repulsive forces, whereas the parameter sign(*λ*_2_) *ρ* < 0 illustrates attractive interactions, between interacting components. If parameter sign(*λ*_2_) *ρ* = 0, then van der Waals interactions are in adducts.^38,39^

**Fig. 5:**
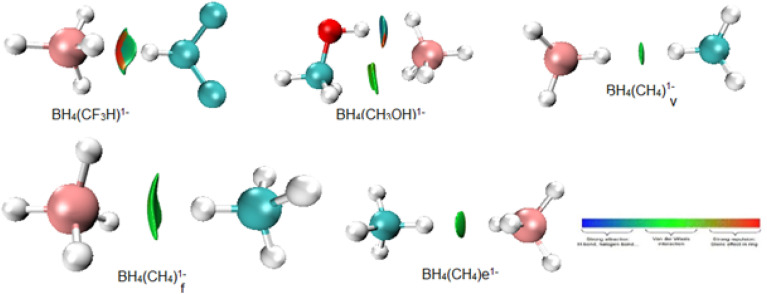
NCI analysis of BH_4_(L)^1−^ adducts.

**Fig. 6:**
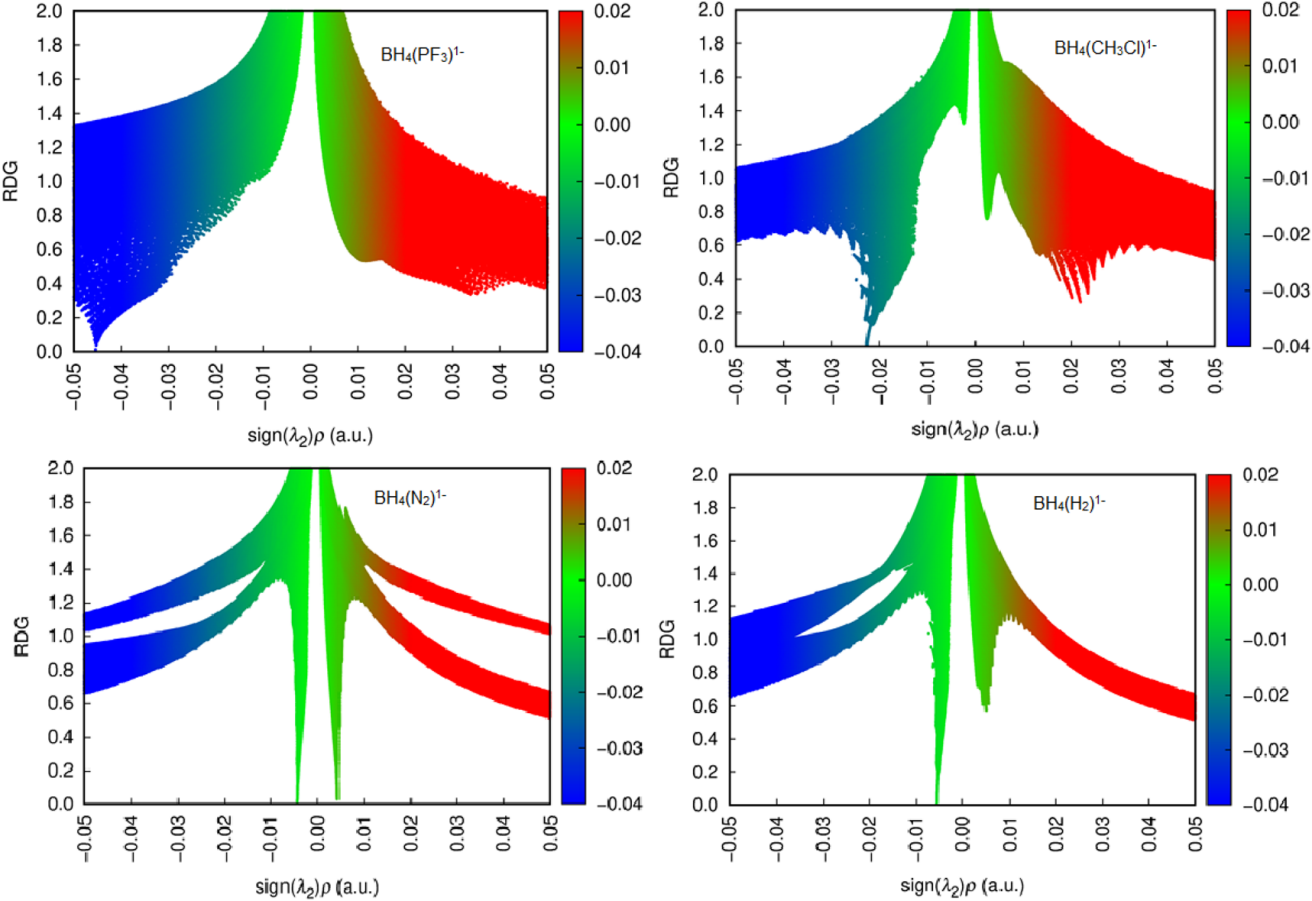
3D iso-surface of BH_4_(L)^1−^ adducts.

Notably, different types of NCIs, including weak van der Waals forces, attractive interactions, and steric repulsion forces, were observed in BH_4_(L)^1−^ complexes (Fig. 5 and S1). The 3D color-filled RDG isosurfaces shown in Fig. 6 and S2 also illustrate the steric repulsions, noncovalent bonds, and weak van der Waals forces between BH_4_^1−^ and L molecules. In NCI 3D images, the *λ*_2_ sign has been used to distinguish between attractive and repulsive interactions based on a particular color. In the context of NCI plots, blue surfaces represent strong attractive interactions, weak attractive interactions (weak van der Waals forces) are typically shown in green, while repulsive interactions are depicted in red, as shown in Fig. 6 and S2. The density and area of green areas between BH_4_^1−^ and L molecules were not identical, which indicated that the interactions of BH_4_^1−^ with various molecules occurred at different energies.

## Conclusions

The interaction between BH_4_^1−^ with 25 small molecules (L) was investigated. Our results provided several interesting insights into the characteristics of BH_4_^1−^ in BH_4_(L)^−1^ adducts. In addition to its vertices, edges, and faces, BH_4_^1−^ can act as an electron donor toward electron acceptor species. Its σ-holes may serve as electron acceptors for electron donor species to form BH_4_(L)^1−^ aggregates. For face-centered interactions (which arise from the contribution of the σ-holes in B–H bonds), most variations were observed for the bond length and stretching vibrational frequency of the B–H bond involved in intermolecular interactions with counterpart molecules. These variations occurred as a contraction along with a blue shift for this bond. For vertex and edge interactions, most variations were elongation and a red shift for B–H bonds during intermolecular interactions.

## Conflicts of interest

There is no conflict of interest.

## Supplementary Material

RA-015-D5RA05000F-s001

## Data Availability

The data supporting the conclusions reached from our study are included in the article. Supplementary information: Table S1, the *XYZ* coordinates for the gas phase of optimized structures; Table S2, the SE^un^, ΔZPE, SE^ZPE^, BSSE, SE^ZPE+BSSE^, Δ*H*, and Δ*G* of adducts; Fig. S1, the NCI analysis and Fig. S2, 3D iso-surface of BH_4_(L)^1−^ adducts. See DOI: https://doi.org/10.1039/d5ra05000f.
