# Supplementary material for: Ab initio insights into the face, edge, and vertex interactions of BH41− with electron-accepting molecules
Source: RSC Adv. 2025 Oct 15;15(46):38729–42. doi: 10.1039/d5ra05000f (PMC12526028; doi:10.1039/d5ra05000f)
Supplement: RA-015-D5RA05000F-s001 [file RA-015-D5RA05000F-s001.pdf]

Supplementary Information (SI) for RSC Advances.
This journal is © The Royal Society of Chemistry 2025

Ab Initio Insights into Face, Edge, and Vertex Interactions of BH_4^{1-} with Electron-Accepting Molecules

Abedien Zabardasti*, Mohammad Solimannejad*, Mohammad N. AL-Baiati, Maryam Salehnassaj

Table S1. XYZ Coordinates for the gas phase optimized structures at MP2/aug-cc-pVDZ

$BH_4(H_2)_f^{1-}$				$BH_4(H_2)_v^{1-}$			
B	0.58247900	0.00015200	0.00016900	B	-0.71298500	-0.00000200	-0.00000200
H	0.15779600	-0.25249900	1.14652300	H	-1.12742200	1.11472600	-0.37949600
H	0.16735000	-0.87063000	-0.79276900	H	-1.12624700	-0.88618300	-0.77607500
H	1.82970600	0.00420200	0.00380200	H	0.53405400	0.00062800	0.00051600
H	-2.99298300	-0.00062900	-0.00095100	H	-1.12738500	-0.22917700	1.15505100
H	0.15790000	1.11832400	-0.35833100	H	2.82591200	0.00007400	0.00006300
H	-2.23216500	0.00047200	0.00087800	H	3.58601400	-0.00006000	-0.00004900
$BH_4(CF_3Cl)_v^{1-}$				$BH_4(CF_3H)_f^{1-}$			
B	-3.86211000	-0.02070100	-0.00024900	B	-2.92210600	0.00002500	0.00059400
H	-3.48590000	-0.56856600	1.05496200	H	-2.48420400	0.62690600	0.98643800
H	-3.45440000	-0.68200200	-0.97576000	H	-4.16073100	0.00033100	0.00350300
H	-5.10340400	0.04073700	-0.02386200	H	-2.48900900	0.54074900	-1.03705500
H	-3.38620900	1.13146200	-0.05732900	H	-2.48715600	-1.16843000	0.04931100
C	1.00626100	-0.00480000	-0.00007500	C	0.39546800	-0.00001000	0.00019700
F	1.55051500	1.24084500	-0.00583400	F	0.90857900	-0.67499900	-1.06913400
F	1.49999400	-0.65180200	-1.08821500	F	0.91090300	-0.58814800	1.11869000
F	1.50195600	-0.64256400	1.09267900	F	0.90952700	1.26320100	-0.05043400
Cl	-0.72172300	0.04072700	0.00094200	H	-0.70226000	-0.00010800	0.00154100

$BH_4(CCl_3H)_f^{1-}$

H	-2.74082500	-1.15017600	0.20688900
H	-2.74157800	0.75282900	0.89372600
H	-2.73723300	0.39611800	-1.09762100
H	-4.41443400	-0.00008600	-0.00287300
C	-0.00070400	0.00000200	0.00004500
H	-1.10515200	-0.00015600	-0.00005700
Cl	0.58109800	-0.57486400	1.58951800
Cl	0.58077500	1.66410500	-0.29689800
Cl	0.58111900	-1.08909500	-1.29265300
B	-3.17748100	-0.00020800	0.00004800

$BH_4(CH_3OH)_v^{1-}$

B	2.14864700	-0.11742500	0.00000900
H	1.72363700	-0.69614900	-1.01581500
H	1.72511600	-0.69488000	1.01716100
H	3.38864400	-0.09592800	-0.00087000
H	1.73951900	1.06609600	-0.00040500
O	-0.91288300	0.80108900	0.00004200
H	0.07140200	0.80618100	-0.00041000
C	-1.30354900	-0.56834200	-0.00000100
H	-2.40608400	-0.59355000	0.00070100
H	-0.93115200	-1.10146900	-0.89247200
H	-0.92995800	-1.10183900	0.89174000

$BH_4(HCN)_f^{1-}$

B	2.30227500	0.00006300	0.00007100
H	1.86430800	-0.08909600	1.16405700
H	3.53879700	-0.00072500	-0.00024300
H	1.86354700	-0.96298600	-0.65925500
H	1.86475000	1.05303100	-0.50439300
N	-2.14533900	0.00013500	0.00012100
C	-0.96078200	-0.00016900	-0.00015200
H	0.13929400	-0.00047500	-0.00044900

$BH_4(HCl)_e^{1-}$

B	2.23170700	-0.03581900	0.00002300
H	2.14715500	-0.71644500	1.02390600
H	2.14892200	-0.71995100	-1.02159800
H	1.27540200	0.80455300	-0.00219700
H	3.23557600	0.68524900	-0.00009700
H	0.20988600	0.27994600	-0.00031900
Cl	-1.18679300	-0.00907400	0.00001100

$BH_4(ClCN)_v^{1-}$

B	-3.10025900	0.00048500	-0.00042700
H	-2.67657000	-0.62094900	-0.99567100
H	-4.34004900	0.00064100	0.00067000
H	-2.67497100	-0.55057500	1.03482500
H	-2.67564000	1.17277100	-0.04136000
N	2.77827300	0.00062400	-0.00048000

$BH_4(FCN)_v^{1-}$

B	2.92195400	-0.00031000	0.00024600
H	2.50239700	-0.90924400	-0.74610100
H	4.16709800	-0.00068200	0.00058500
H	2.50290600	1.10060400	-0.41401700
H	2.50207000	-0.19191000	1.16050700
N	-2.52504300	-0.00050700	0.00041100

C	1.58540400	0.00007400	-0.00014500	C	-1.33635300	0.00005500	-0.00006800
Cl	-0.06422400	-0.00053700	0.00046500	F	-0.06564700	0.00066700	-0.00051900
$BH_4(HF)_e^{1-}$				$BH_4(SHF)_v^{1-}$			
B	-1.58613600	0.01214200	-0.00001800	B	2.55745300	0.13372000	0.06674900
H	-1.04691900	1.12853300	-0.00242200	H	2.44678200	1.33453100	0.25705100
H	-2.27021400	-0.13328600	-1.02294300	H	3.14282700	-0.17849800	-0.96436100
H	-0.71532700	-0.88886700	0.00160400	H	2.84101400	-0.53262000	1.04964200
H	-2.26979000	-0.12909100	1.02377300	H	1.32111300	-0.32587900	-0.29304100
H	0.56254300	-0.10880800	0.00020600	S	-0.22031400	-0.22279800	-0.04025200
F	1.51893200	0.00786800	-0.00001400	F	-2.10668200	0.16865400	0.06356600
				H	-0.05384100	1.08075300	-0.31109600
$BH_4(HOBr)_v^{1-}$				$BH_4(HOCl)_v^{1-}$			
B	-2.88968700	0.08550800	0.01270700	B	2.73392100	0.03632600	-0.00147900
H	-3.03116800	1.14037900	-0.59080600	H	1.33989500	-0.02906200	0.00530900
H	-1.54968500	-0.13103100	-0.03678400	H	3.02002100	-1.05735800	-0.46720400
H	-3.11552200	0.13144500	1.21458000	H	2.92752100	1.00094300	-0.72737200
H	-3.30695500	-0.91150000	-0.56474300	H	2.97135300	0.19784400	1.18700900
O	2.22645000	-0.00773900	0.01865600	Cl	-0.21061100	-0.03877700	0.00138800
H	2.36104800	0.95550900	-0.02018400	O	-2.24853900	-0.06700000	-0.00169400
Br	0.15083200	-0.04429800	-0.00613800	H	-2.35969000	0.90120900	-0.00039600
$BH_4(H_2O)_v^{1-}$				$BH_4(SH_2)_v^{1-}$			
B	-1.64826200	0.01146900	0.00426400	B	-2.36763000	0.01068300	0.00568400
H	-1.11732400	1.01935700	-0.50262800	H	-2.29652400	0.25046000	1.22303900
H	-1.76072600	0.16735500	1.23161400	H	-3.53892800	-0.23207500	-0.32711800
H	-2.75858600	-0.18395900	-0.51568400	H	-1.92731300	0.97837800	-0.64255500
H	-0.93015600	-0.99165400	-0.21683100	H	-1.66780300	-0.99758400	-0.24973500
O	1.59304100	-0.06426400	0.00632800	S	1.26921300	-0.06110200	0.00383700
H	0.68284900	-0.43238800	-0.04535800	H	-0.07727000	-0.34261700	-0.07401600
H	1.38092900	0.87805700	-0.02306100	H	1.03858700	1.26766300	-0.01942200

$BH_4(SF_2)_v^{1-}$

B	2.60433800	-0.24516200	0.00022800
H	2.35323800	-1.44585600	0.00054700
H	3.09794400	0.17992300	1.03665200
H	3.09954900	0.17900300	-1.03581300
H	1.40010200	0.32543500	-0.00086000
F	-1.90865800	-0.59672500	0.00025000
F	-0.46709000	1.35749700	0.00002700
S	-0.09942500	-0.30372800	-0.00026000

$BH_4(PHF_2)_v^{1-}$

B	2.66591700	-0.23948700	0.06442100
H	3.47492200	0.05703300	-0.81802000
H	2.32298700	-1.42802200	0.00499300
H	3.04580900	0.07770700	1.19477900
H	1.61839000	0.44295800	-0.18463700
P	-0.18298300	-0.24748000	-0.21131800
F	-0.51692400	1.37923500	0.05470700
F	-1.84365500	-0.67552100	0.11543500
H	0.19825200	-0.57346200	1.11928100

$BH_4(PH_3)_v^{1-}$

B	2.38590300	-0.00089100	0.00687300
H	3.59273900	-0.11689100	-0.27621400
H	2.10726200	-0.72128900	0.98634600
H	2.12828200	1.18929900	0.28457200
H	1.68939800	-0.34928800	-0.97014200
P	-1.22256300	0.00397800	-0.12537700
H	-0.65805500	-1.15172200	0.48174300
H	-0.25857300	0.89171200	0.42429500
H	-2.19211700	0.20296300	0.91569000

$BH_4(PH_2F)_v^{1-}$

B	-2.61898600	0.11954900	0.04740300
H	-3.40685300	-0.82608300	0.13839400
H	-2.80253700	0.79739400	-0.96614800
H	-2.56852000	0.79923200	1.07303500
H	-1.47830100	-0.46086800	-0.11646200
P	0.32218600	-0.23549400	-0.13520400
F	2.05138200	0.19352800	0.07992800
H	-0.04707900	1.12258800	-0.32748900
H	0.10298600	-0.23935200	1.27035900

$BH_4(PF_3)_v^{1-}$

B	-2.71606500	0.00017300	-0.30996100
H	-2.61640900	-1.02181900	-1.00136500
H	-2.62045200	1.01677400	-1.00978400
H	-3.73508200	0.00099800	0.38965800
H	-1.73521100	0.00531200	0.48652200
P	0.14697900	0.00015000	-0.33016500

$BH_4(CH_4)_e^{1-}$

H	2.87917700	0.98148200	-0.00645600
H	1.39049600	0.04120000	1.01955200
H	1.38406300	0.03266900	-1.01530200
H	2.77499300	-1.05511300	0.00239800
C	-1.89579500	-0.00007500	0.00024900
H	-2.26823500	0.95653300	-0.39567100

F	0.31272400	-1.23741500	0.75157900	H	-2.28354600	-0.14403900	1.01971000
F	1.82401000	-0.00290200	-0.65172400	H	-2.25416800	-0.81849800	-0.64175800
F	0.31691100	1.23983100	0.74872700	H	-0.79600000	0.00643800	0.01619000
				B	2.10959800	-0.00004500	-0.00003200
$BH_4(CH_4)_v^{1-}$				$BH_4(CH_4)_f^{1-}$			
H	-1.14377000	0.00220300	-0.00195200	B	-1.98341900	0.00000000	0.00000000
H	-2.80095900	-0.61095600	1.00806600	H	-1.55932700	-0.16615100	1.16243800
H	-2.80567600	1.17682500	0.02536800	H	-3.22972000	-0.00070800	0.00123100
H	-2.80421200	-0.56811100	-1.03154600	H	-1.56161300	1.09049800	-0.43774000
C	2.14917000	0.00000500	-0.00000400	H	-1.56075500	-0.92364200	-0.72592100
H	2.52102100	-0.75092500	-0.71232500	C	1.78100600	-0.00000300	0.00000500
H	2.52051500	-0.24193600	1.00654400	H	0.68136800	-0.00072400	0.00079600
H	2.52193000	0.99216100	-0.29368500	H	2.15464000	-0.96138400	0.38302100
H	1.04947000	0.00067600	-0.00051000	H	2.15282100	0.14911200	-1.02472200
B	-2.39066800	0.00000700	0.00001300	H	2.15364500	0.81301200	0.64086900
$BH_4(CO)_v^{1-}$				$BH_4(N_2)_v^{1-}$			
B	-2.04956200	0.01752700	-0.00010500	B	-2.08808000	0.00433900	0.00002600
H	-1.73717000	1.22609500	-0.01084500	H	-1.66660200	1.17915800	-0.00054800
H	-3.29035800	-0.10243800	0.00905600	H	-3.33523500	0.00191600	0.00020000
H	-1.57943500	-0.53527900	-1.01613000	H	-1.66593100	-0.58185000	-1.01799800
H	-1.56692200	-0.52023600	1.01817200	H	-1.66540800	-0.58102400	1.01826500
C	1.24049900	-0.66453000	0.00001800	N	1.32412500	-0.56858600	0.00000200
O	1.37233800	0.47892500	0.00002100	N	1.35781500	0.56288700	-0.00000800

Table S2: The SE^{un}, ΔZPE, SE^{ZPE}, BSSE, $SE^{ZPE+BSSE}$, ΔH, and ΔG in $kcal.mol^{-1}$ calculated at MP2/aug-cc-

Adduct	SE^{un}	ΔZPE	SE^{ZPE}	BSSE	$SE^{ZPE+BSSE}$	ΔH	ΔG
$BH_4(ClCN)_v^{1-}$	-14.17	0.45	-13.72	0.98	-12.74	-13.52	-8.78
$BH_4(BrCN)_v^{1-}$	-19.79	1.32	-18.47	2.19	-16.28	-18.49	-12.10
$BH_4(CF_3Cl)_v^{1-}$	-8.74	0.67	-8.07	0.94	-7.13	-8.03	-2.02
$BH_4(FCN)_v^{1-}$	-7.51	0.9	-6.61	0.65	-5.96	-6.34	-2.74
$BH_4(CO)_v^{1-}$	-3.34	0.87	-2.47	0.60	-1.87	-2.31	2.10
$BH_4(N_2)_v^{1-}$	-2.59	0.47	-2.12	0.73	-1.39	-1.90	1.35
$BH_4(H_2O)_v^{1-}$	-13.41	2.25	-11.16	0.93	-10.23	-11.65	-6.02
$BH_4(CF_3H)_f^{1-}$	-15.75	1.2	-14.55	1.55	-13	-14.35	-7.67
$BH_4(CH_3OH)_v^{1-}$	-14.48	1.57	-12.91	1.23	-11.68	-12.93	-6.89
$BH_4(HCl)_e^{1-}$	-18.83	1.58	-17.25	1.37	-15.88	-18.19	-11.64
$BH_4(HCN)_f^{1-}$	-19.63	1.35	-18.28	1.18	-17.1	-18.38	-12.77
$BH_4(HF)_e^{1-}$	-19.50	2.39	-17.11	1.04	-16.07	-17.94	-12.07
$BH_4(CH_4)_f^{1-}$	-3.08	0.73	-2.35	0.65	-1.7	-1.94	1.94
$BH_4(CH_4)_e^{1-}$*	-2.74	0.65	-2.09	0.61	-1.48	-2.16	2.11
$BH_4(CH_4)_v^{1-}$	-2.12	0.6	-1.52	0.58	-0.94	-0.90	-1.08
$BH_4(HOBr)_v^{1-}$	-24.32	0.56	-23.76	2.94	-20.82	-23.64	-18.65
$BH_4(HOCl)_v^{1-}$	-19.05	0.36	-18.69	2.08	-16.61	-19.29	-11.15
$BH_4(SF_2)_v^{1-}$	-26.17	1.89	-24.28	2.70	-21.58	-24.58	-16.98
$BH_4(SFH)_v^{1-}$	-28.55	1.98	-26.57	2.70	-23.87	-26.82	-19.52
$BH_4(SH_2)_v^{1-}$	-11.11	1.57	-9.54	1.11	-8.43	-9.79	-3.71
$BH_4(PH_3)_v^{1-}$	-5.92	1.21	-4.71	0.83	-3.88	-4.59	0.86
$BH_4(PH_2F)_v^{1-}$	-20.43	2.07	-18.36	2.00	-16.36	-18.67	-10.79
$BH_4(PHF_2)_v^{1-}$	-19.54	1.97	-17.57	2.17	-15.4	-17.85	-9.65
$BH_4(PF_3)_v^{1-}$	-16.45	1.64	-14.81	2.40	-12.41	-14.92	-7.46
$BH_4(H_2)_f^{1-}$	-2.15	1.58	-0.57	0.30	-0.27	1.12	-4.86
$BH_4(H_2)_v^{1-}$	-1.56	1.55	-0.01	0.33	0.32	1.79	-5.44
$BH_4(H_2)_e^{1-}$*	-1.94	1.55	-0.39	0.30	-0.09	0.78	-4.01
$BH_4(CCl_3H)_f^{1-}$	-18.30	1.25	-17.05	2.90	-14.15	-16.88	-9.81

pVDZ.

- $BH_4(CH_4)_e^{1-}$* (v_1 = -48(1)) and $BH_4(H_2)_e^{1-}$* (v_1 = -29(1)) are optimized nonlocal structures.
- SE^{un} uncorrected stabilization energies, ΔZPE zero point corrections, SE^{ZPE} zero point corrected stabilization energies, $SE^{ZPE+BSSE}$ zero point and counterpoise corrected stabilization energies.

$SE^{un} = E_{complex} – (E_{BH4} + E_L)$, $\Delta ZPE = ZPEcomplex – (ZPE_{BH4}^- + ZPE_L)$, $SE^{ZPE} = SE^{un} + \Delta ZPE$,

$SE^{ZPE+BSSE} = SE^{un} + (\Delta ZPE + BSSE)$; $E_{complex,}$ E_{BH4} and E_L are internal energies of related compounds.

Fig. S1: The NCI analysis of $BH_4(L)^{1-}$ adducts.

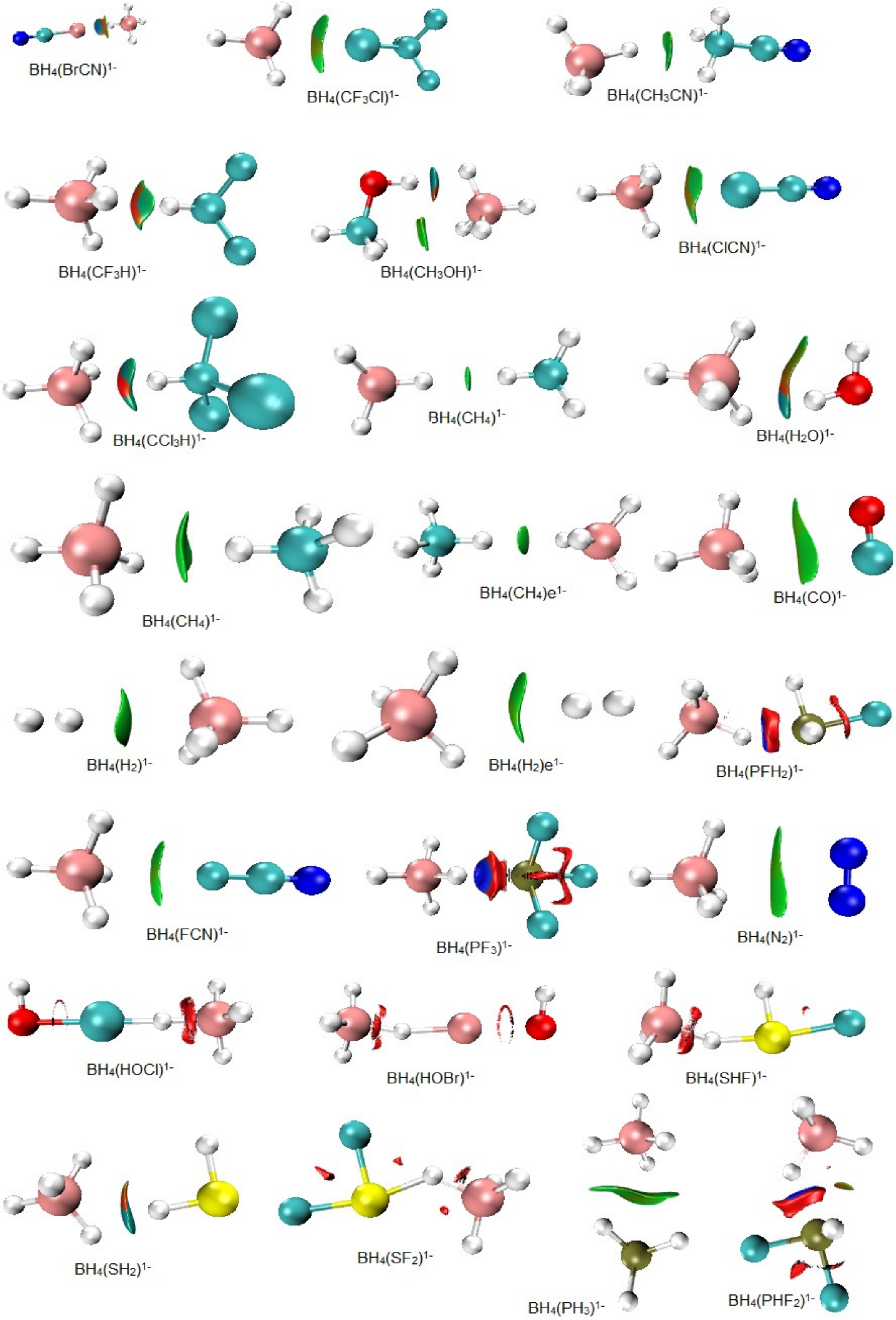

Fig. S2: 3D iso-surface of $BH_4(L)^{1-}$ adducts.

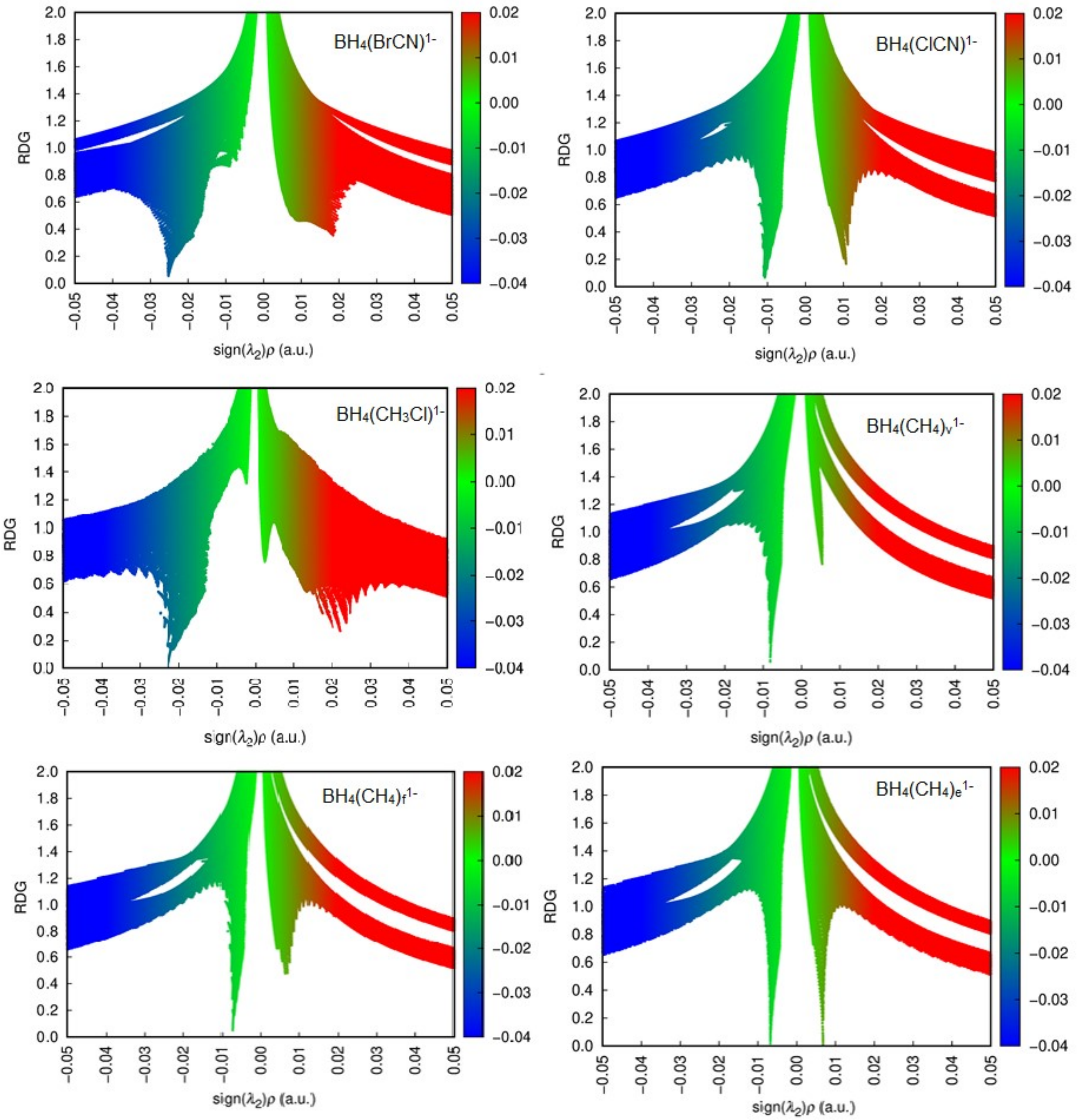

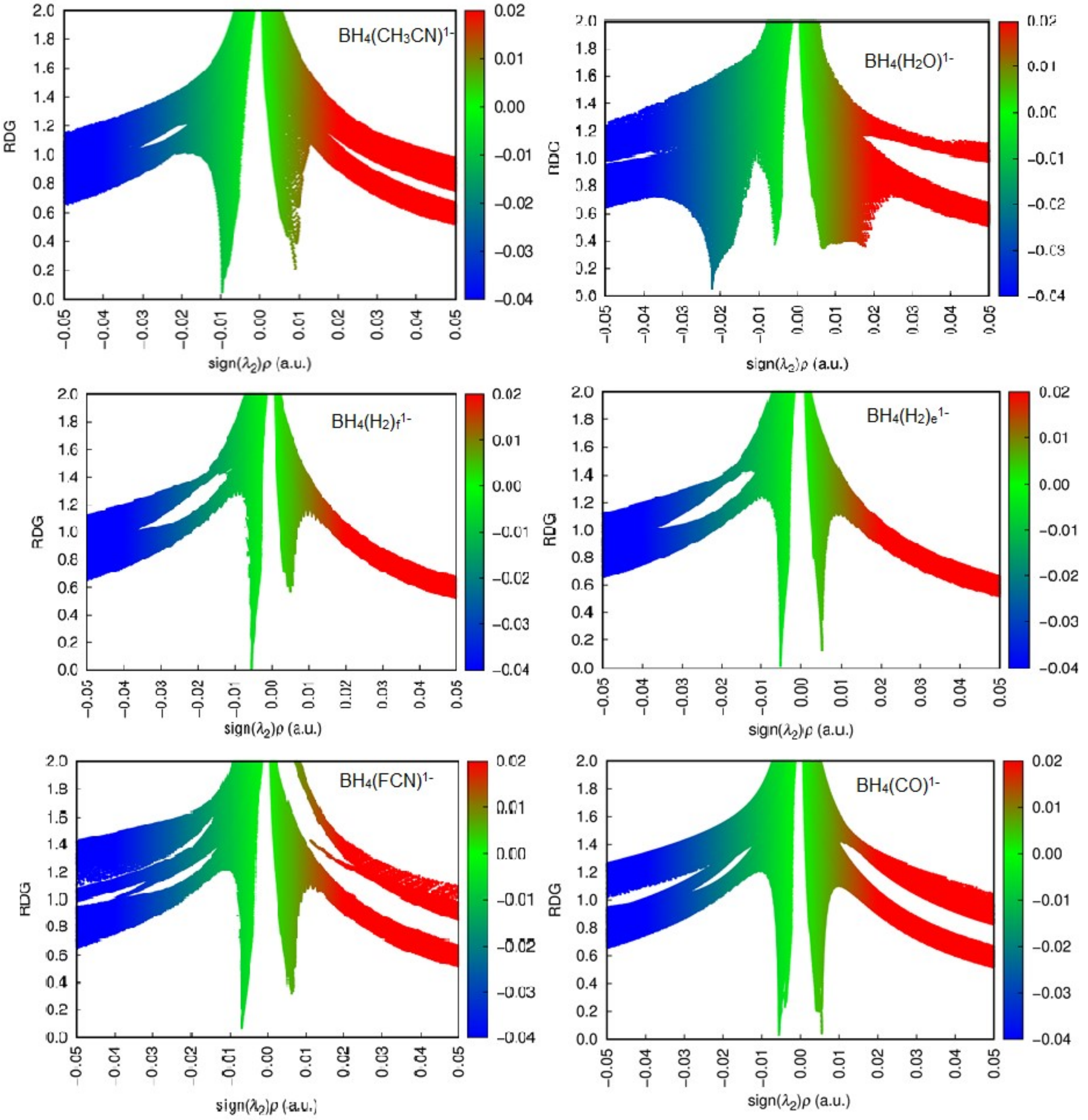
BH4(CH3CN)1-
BH4(H2O)1-
BH4(H2)f1-
BH4(H2)e1-
BH4(FCN)1-
BH4(CO)1-
RDG
sign(λ2)ρ (a.u.)
2.0
1.8
1.6
1.4
1.2
1.0
0.8
0.6
0.4
0.2
0.0
−0.05
−0.04
−0.03
−0.02
−0.01
0.00
0.01
0.02
0.03
0.04
0.05
−0.04
−0.03
−0.02
−0.01
0.00
0.01
0.02

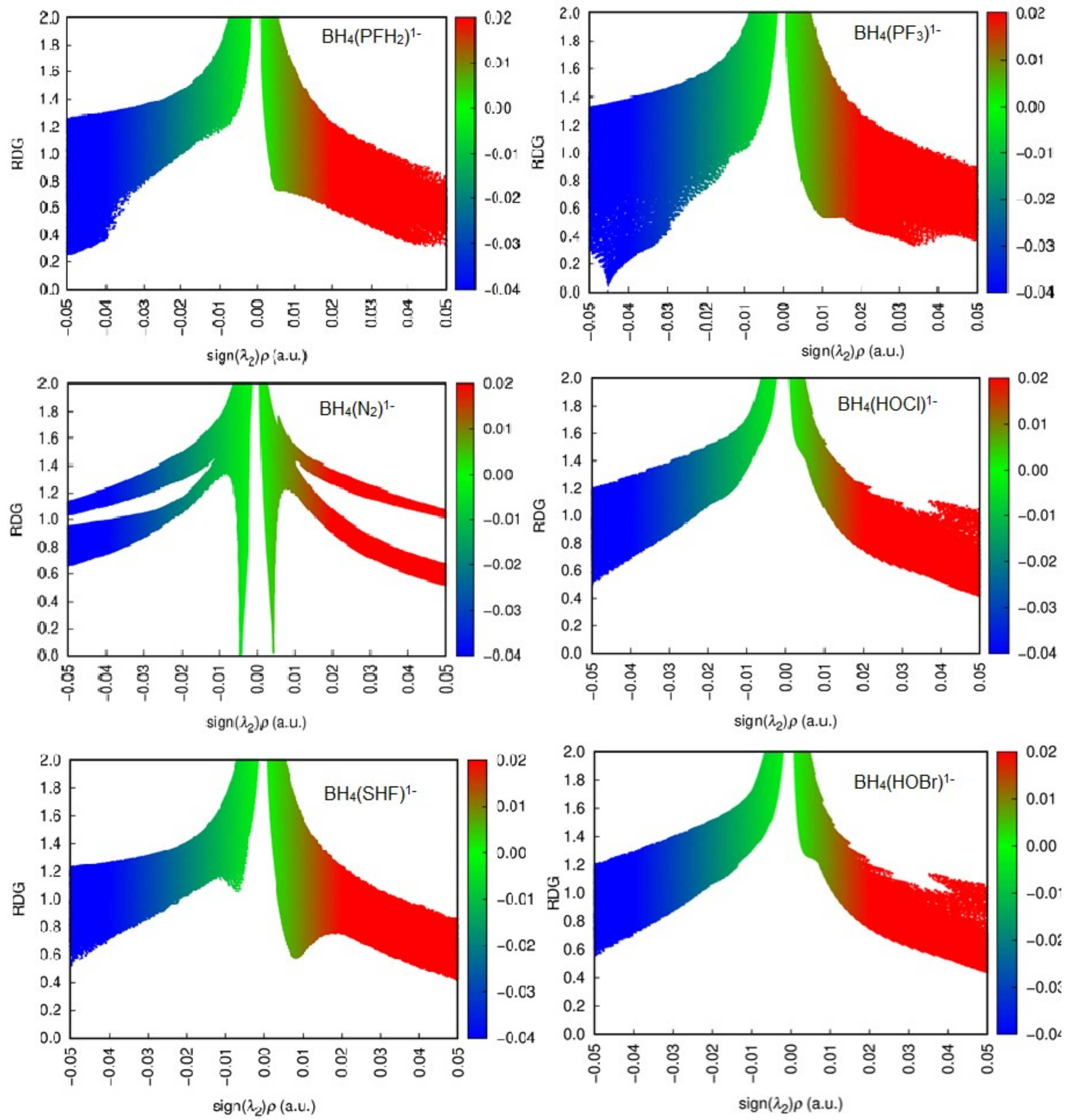
BH4(PFH2)1-
BH4(PF3)1-
BH4(N2)1-
BH4(HOCl)1-
BH4(SHF)1-
BH4(HOBr)1-
RDG
sign(λ2)ρ (a.u.)

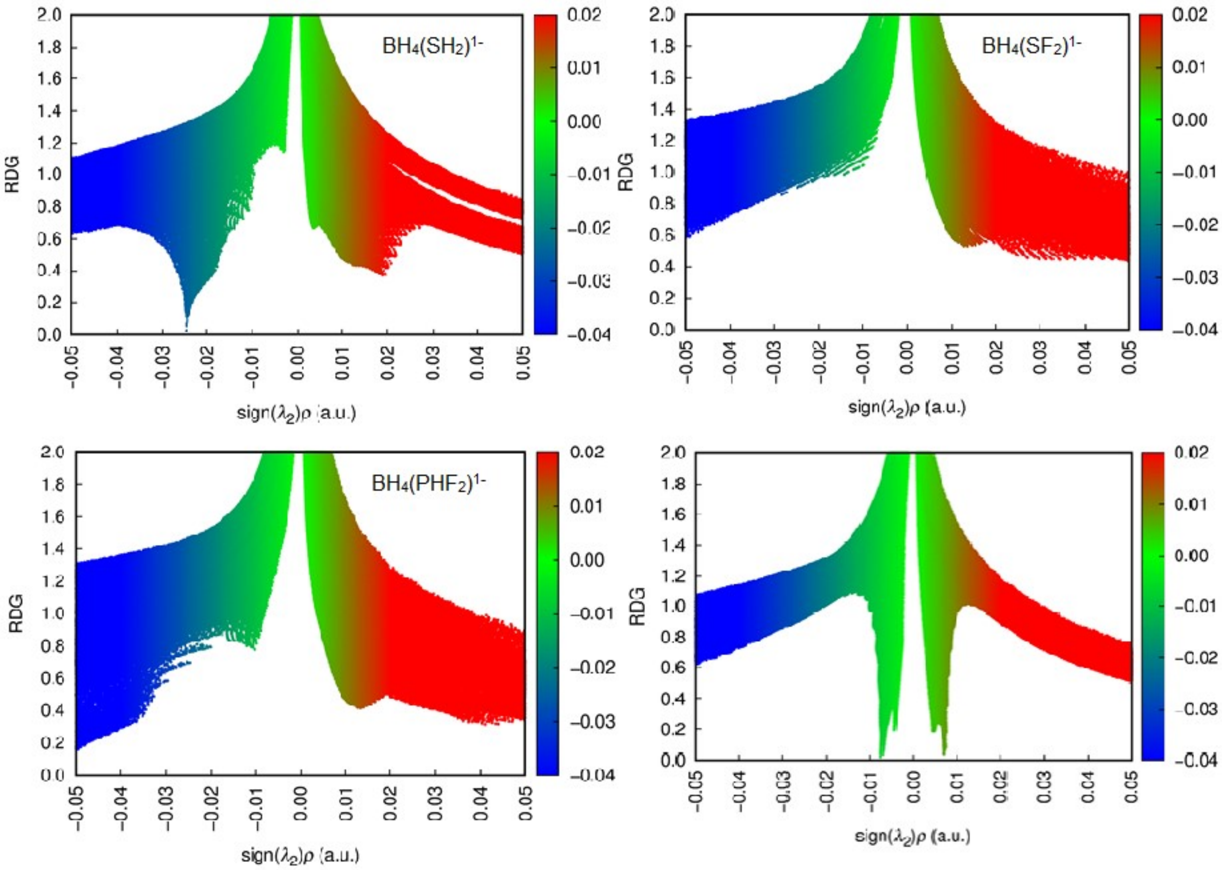

BH4(SH2)1-
BH4(SF2)1-
BH4(PHF2)1-
RDG
sign(λ2)ρ (a.u.)
